# Systematic Full-Cycle Engineering Microbial Biofilms to Boost Electricity Production in *Shewanella oneidensis*

**DOI:** 10.34133/research.0081

**Published:** 2023-03-15

**Authors:** Feng Li, Rui Tang, Baocai Zhang, Chunxiao Qiao, Huan Yu, Qijing Liu, Junqi Zhang, Liang Shi, Hao Song

**Affiliations:** ^1^ Frontiers Science Center for Synthetic Biology (Ministry of Education), and Key Laboratory of Systems Bioengineering, Tianjin University, Tianjin 300072, China.; ^2^ Collaborative Innovation Center of Chemical Science and Engineering (Tianjin), School of Chemical Engineering and Technology, Tianjin University, Tianjin 300072, China.; ^3^ Department of Biological Sciences and Technology, School of Environmental Studies, China University of Geoscience in Wuhan, Wuhan, Hubei 430074, China.

## Abstract

Electroactive biofilm plays a crucial rule in the electron transfer efficiency of microbial electrochemical systems (MES). However, the low ability to form biofilm and the low conductivity of the formed biofilm substantially limit the extracellular electron transfer rate of microbial cells to the electrode surfaces in MES. To promote biofilm formation and enhance biofilm conductivity, we develop synthetic biology approach to systematically engineer *Shewanella oneidensis*, a model exoelectrogen, via modular manipulation of the full-cycle different stages of biofilm formation, namely, from initial contact, cell adhesion, and biofilm growth stable maturity to cell dispersion. Consequently, the maximum output power density of the engineered biofilm reaches 3.62 ± 0.06 W m^−2^, 39.3-fold higher than that of the wild-type strain of *S. oneidensis*, which, to the best our knowledge, is the highest output power density that has ever been reported for the biofilms of the genetically engineered *Shewanella* strains.

## Introduction

Electroactive biofilm formed by an intimate interfacing biocatalytic machinery of electroactive cells with electrodes plays a vital role in determining the performance of a wide variety of microbial electrochemical systems (MES), which are sustainable technologies for energy and chemicals production [1–5]. Extracellular electron transfer (EET) rate underlies the efficiency of electroactive biofilm [6,7]. However, the EET rate was restricted by the limited ability of biofilm formation and low conductivity of biofilm, rendering applications of MES unpractical [3,6,8–10].

Substantial efforts have been made to elucidate the fundamental molecular mechanisms of EET in biofilm and to optimize EET rate from the perspectives of engineering electroactive cells, optimizing electrode materials, and bioelectrochemical reactor design [7,8,11–20]. *Shewanella oneidensis* MR-1 is one of the most well-studied metal-reducing exoelectrogens, which can conduct EET via *c*-type cytochromes (*c*-Cyts) and flavins as electron shuttles or bound cofactors for outer-membrane *c*-Cyts (OM *c*-Cyts) [9,21]. On the basis of these 2 underlying EET mechanisms, a number of synthetic biology strategies were developed to enhance the EET rate in *S. oneidensis*, including broadening feedstock spectrum [22,23], increasing intracellular electron generation [7,12,24], optimizing conductive *c*-Cyts systems [14,25–27], and accelerating biosynthesis and secretion of electron shuttles [28,29], which mainly focus on increasing electron generation and promoting EET at a single-cell level. However, improvement of the EET rate between biofilm and electrode at a multicellular level has not been fully explored.

Electroactive biofilms are the conductive microbial communities embedded in a structured, complex, self-produced polymeric matrix on electrode surfaces, which deliver intracellular electrons onto electrode surfaces [30–33]. Previous efforts focused on improving the understanding of biofilm architecture and mechanisms by means of genetic engineering and random transposon insertion methods [34–42]. However, it remains unclear whether manipulation of the full-cycle biofilm formation process could facilitate the thickness and conductivity of electroactive biofilm.

**Fig. 1.**
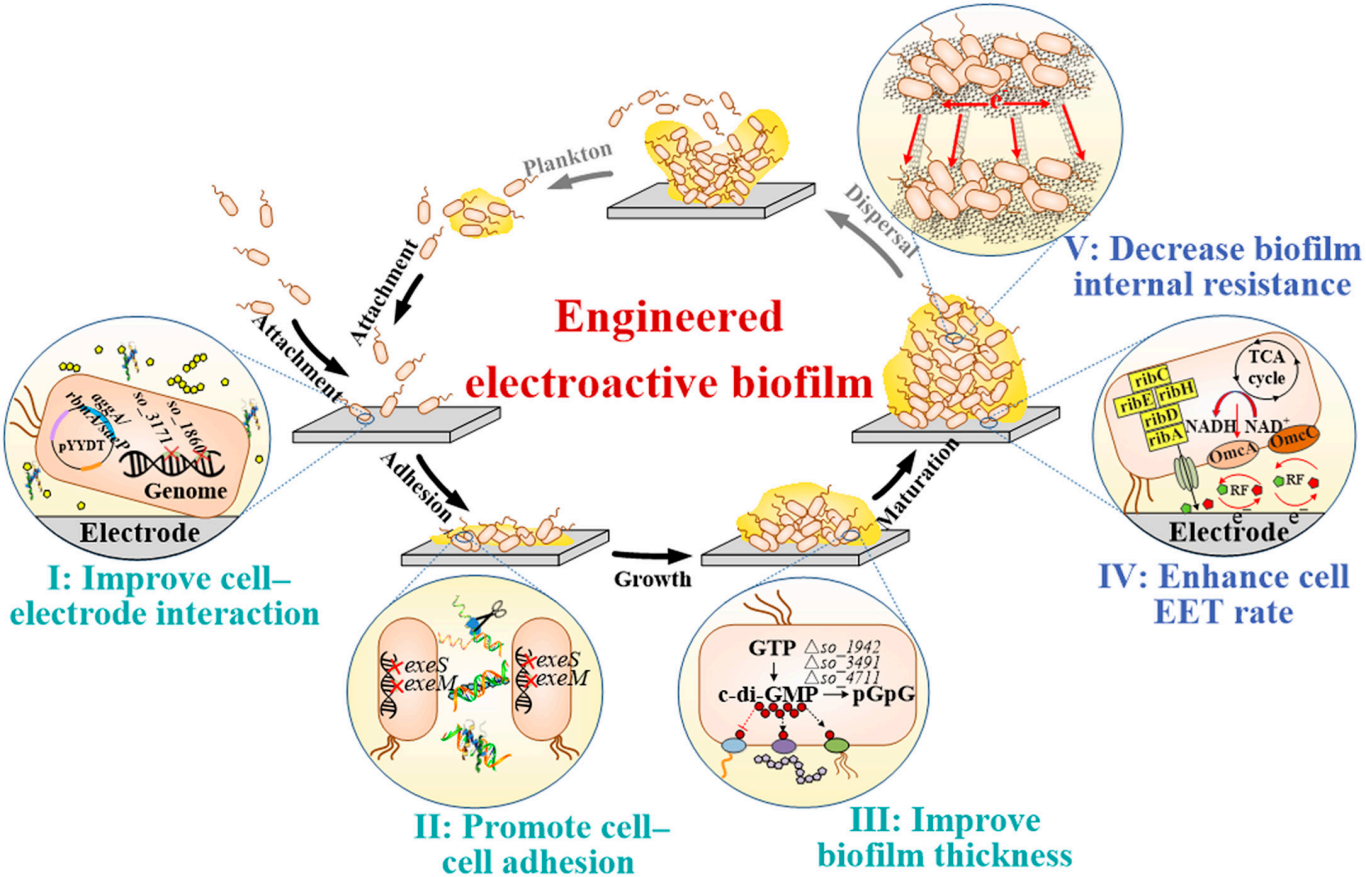
Schematic illustration of modular full-cycle biofilm engineering to promote biofilm formation and conductivity of *S. oneidensis.* We engineered *S. oneidensis*, a model exoelectrogen, using synthetic biology modular strategy to promote biofilm formation and to enhance biofilm conductivity via regulating the full-cycle biofilm formation process, namely, the initial contact (I), cell adhesion (II), biofilm growth (III), stable maturity stage (IV), and dispersion stage (V). To promote biofilm formation ability, we enhanced the cell coverage on electrode surfaces by engineering the initial contact stage (I), promoted cell−cell adhesion in the adhesion stage (II), and increased the vertical extension of biofilm to reinforce the 3-dimensional (3D) structure formation in the biofilm growth stage (III). To increase biofilm conductivity, the synthesis of outer-membrane *c*-type cytochromes (*c*-Cyts) and riboflavin were strengthened to enhance EET rate of each cell of natural electroactive biofilm in the stable maturity stage (IV). Then, an engineered rGC/SE^r^GC3F 3D self-assembled artificial electroactive biofilm was further constructed to increase the electron collection capacity and reduce internal resistance of artificial electroactive biofilm in maturity dispersion stage (V). TCA, tricarboxylic acid cycle; NAD^+^/H, nicotinamide adenine dinucleotide.

To overcome the limited biofilm-forming ability and low biofilm conductivity of *S. oneidensis*, we engineered the formation, maturation, and dispersion of biofilm, a developmental and cycling process initiated by planktonic (free-living) microorganisms forming aggregates and/or transitioning to a surface-associated lifestyle undergoing 5 stages, namely, the initial contact stage (I), cell adhesion stage (II), biofilm growth stage (III), stable maturity stage (IV), and dispersion stage (V) (Fig. 1) [43–46]. In this study, we carried out synthetic biology strategies to engineer the initial contact, adhesion, growth, and stable maturity in the full-cycle biofilm development process to promote biofilm-forming ability and enhance the conductivity of *S. oneidensis* biofilm. To improve the biofilm-forming ability, we enhanced the affinity and coverage of the cell–electrode interfaces in the initial contact stage via regulating the hydrophobicity of extracellular polysaccharides, promoted the cell–cell adhesion in the adhesion stage via increasing the content of “conductive glue” extracellular DNA (eDNA), and increased the vertical extension of biofilm to reinforce a 3-dimensional (3D) structure in the growth stage via regulating the content of bis-(3-5)-cyclic dimeric guanosine monophosphate (c-di-GMP) to enhance the biofilm thickness. To further improve the biofilm conductivity, the synthesis of OM *c*-Cyts and riboflavin (RF) were strengthened to enhance EET rate of each cell of the natural electroactive biofilm in the stable maturity stage. Then, an engineered *Shewanella*-reduced graphene oxide (rGO)–carbon nanotubes (CNTs) 3D self-assembled conductive hybrid was constructed to increase the electron collection capacity and reduce internal resistance of an artificial electroactive biofilm in the maturity dispersion stage. The EET rate of the engineered biofilm is characterized by the generation of electricity in microbial fuel cells (MFCs). The output power density of the engineered biofilms was as high as 3.62 ± 0.06 W m^−2^. To the best our knowledge, this is the highest output power density for a biofilm of *S. oneidensis*, which is also 39.3-fold of that of *S. oneidensis* MR-1 (Table S1).

## Results and Discussion

### Improving interaction between microbial cells and electrode surfaces

**Fig. 2.**
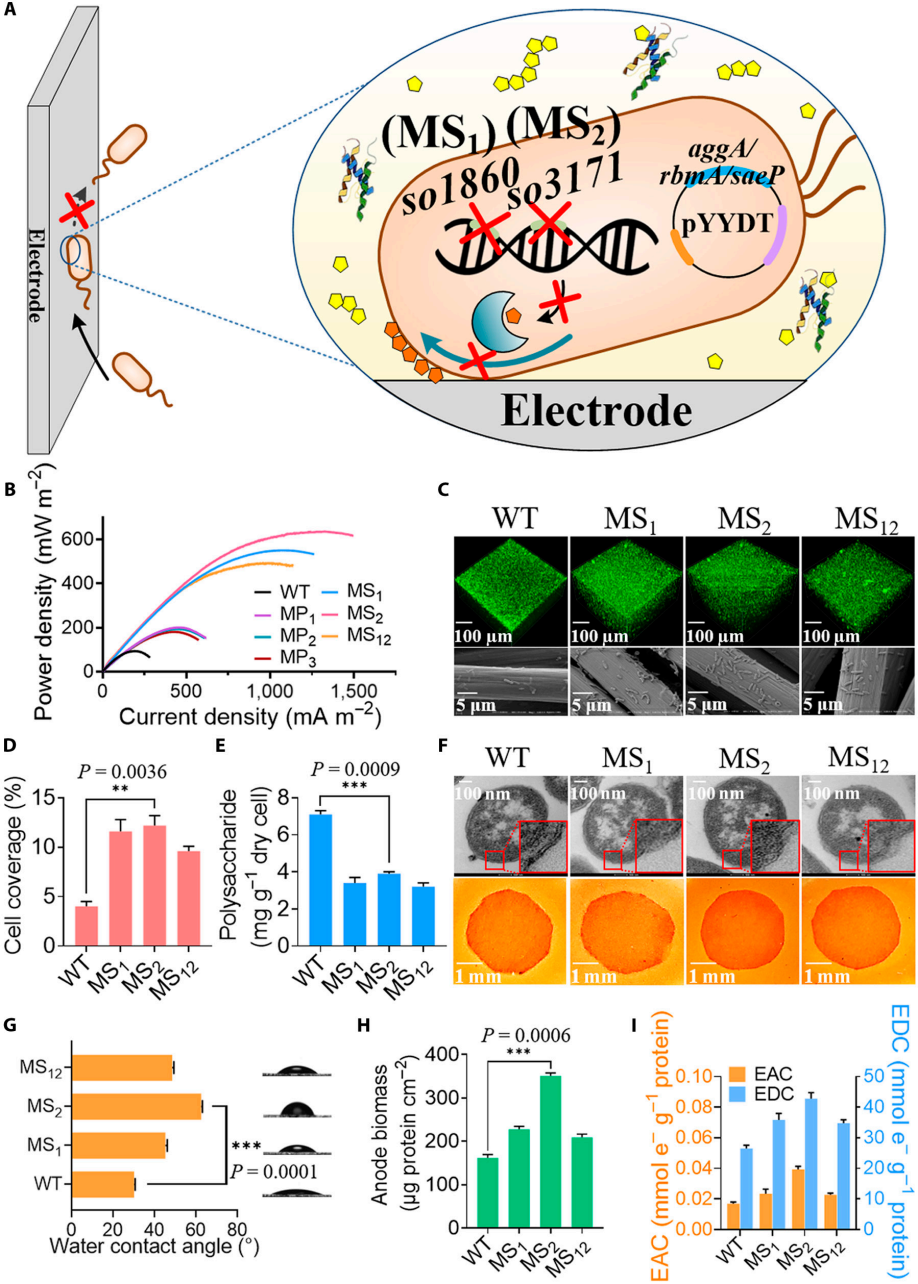
Engineering cell–electrode interface interaction in the initial contact stage (I) of the biofilm formation. (A) Construction of *S. oneidensis* recombinant strains MS_1_ (deleting gene *so1860*), MS_2_ (deleting gene *so3171*), and MS_12_ (deleting 2 genes *so3171* and *so1860* that encode cell surface polysaccharide biosynthesis enzymes), as well as recombinant strains MP_1_, MP_2_, and MP_3_ with individual overexpression of genes *aggA*, *rbmA*, and *saeP* that encode extracellular agglutination protein, adhesive protein, and membrane-attached lipoprotein, respectively. (B) Maximum output power density curves in MFCs of the above *S. oneidensis* recombinant strains. (C) CLSM (top) and SEM (bottom) images of anode biofilms in MFCs equipped with recombinant strains MS_1_, MS_2_, and MS_12_. (D) Cell coverage on electrode surface rate of these recombinant strains. (E) Extracellular polysaccharide contents of anode biofilms in MFCs. (F) TEM images with ruthenium red staining (top) and the colony morphologies (bottom) of recombinant strains MS_1_, MS_2_, and MS_12_. (G) Determination of the surface physicochemical parameters of recombinant strains under the same condition using the water contact angle *θ_W_*. (H) Anode biomass of biofilm formed by *S. oneidensis* recombinant strains in MFCs. (I) EAC and EDC of recombinant strains in MFCs. Data are presented by 3 independent biological replicates as means ± SD.

Interaction between microbial cells and electrode surfaces is the key of the initial contact stage of biofilm formation [47]. As the major constituent of extracellular matrix in biofilms, extracellular polysaccharides and agglutination proteins directly affect the hydrophilicity/hydrophobicity of cell surfaces and cell adhesion to electrode surface, respectively, which subsequently determines the biofilm coverage on the hydrophobic carbon electrode [48–50]. Thus, to improve the initial adhesion of bacterial cells to electrode surfaces, we constructed MS_1_, MS_2_, and MS_12_ strains by deleting the genes *so1860*, *so3171*, and both of them, respectively. According to previous studies on the comparative genomics of the wild-type (WT) *S. oneidensis* [51], the genes *so1860* and *so3171* were found to be involved in the biosynthesis of cell surface polysaccharides. Additionally, we constructed MP_1_, MP_2_, and MP_3_ strains by overexpressing *aggA*, *rbmA*, and *saeP* genes, respectively. The gene *aggA* was found to encode an extracellular agglutination protein that played a key role in cell aggregation and biofilm formation of *S. oneidensis* [52], the gene *rbmA* encoded an adhesive protein involved in facilitating intercellular adhesion during biofilm formation in a typical biofilm pathogen *Vibrio cholerae* [53], while the gene *saeP* encoded a membrane-attached lipoprotein required to anchor a cellular membrane that substantially impact biofilm development in the pathogen *Staphylococcus aureus* (Fig. 2A) [54]. The results showed that output power density decreased in the order of MS_2_ (634 ± 15.6 mW m^−2^) > MS_1_ (549.6 ± 17.2 mW m^−2^) > MS_12_ (490.7 ± 13.7 mW m^−2^) > MP_1_ (200.2 ± 11.6 mW m^−2^) ≈ MP_2_ (192.9 ± 9.4 mW m^−2^) > MP_3_ (180.2 ± 9.3 mW m^−2^) > WT (92 ± 6.5 mW m^−2^) of *S. oneidensis* MR-1 (Fig. 2B). Although the enhancement of extracellular agglutination proteins could increase cell-to-cell and cell-to-surface attachment and facilitate biofilm formation, the large amount of nonconductive proteins accumulated on cell surfaces may inhibit interaction between OM *c*-Cyts, electron shuttles, and electrode surfaces, thus preventing the transportation of electrons from exoelectrogenic cells to electrode surfaces [30,31]. Moreover, the accumulation of agglutination proteins attached on cell surfaces causes strong chemical gradients of nutrients within the biofilm, which would influence bacterial physiology and metabolism, thus limiting the enhancement of EET rate [55,56]. Confocal laser scanning microscope (CLSM) analyses revealed that the biofilm thicknesses of MS_1_, MS_2_, and MS_12_ were 1.4-, 1.7-, and 1.3-fold higher than that of WT, respectively, demonstrating that deletion of these genes enhanced biofilm formation (Fig. 2C). Scanning electron microscope (SEM) analyses also revealed that the cell coverage on the electrode surface by MS_1_, MS_2_, and MS_12_ were 2.9-, 3.0-, and 2.4-fold than WT, respectively (Fig. 2D).

Results of ruthenium red staining (a polysaccharides staining dye that could be visualized by transmission electron microscope (TEM)) and Congo red (a carbohydrate binding dye that inhibited fibril polymerization) assay indicated that the content of cell surface polysaccharides of the recombinant strains with high coverage were impaired (Fig. 2E and F). Analyses of water contact angle (*θ_W_*) and the affinity of cells to hexadecane showed that cell surface hydrophobicity of strain MS_2_ was increased by 2-fold, which increased interaction between the cell and the hydrophobic electrode surface and cell coverage on electrode surfaces (Fig. 2G, Figs. S1 and S2, Table S2, and Eq. 2). These results were in agreement with a previous study that concluded that cell surface hydrophobicity influenced the adhesiveness of *S. oneidensis* cells to graphite felt electrodes [57]. Consequently, the biomass attached on the anode surface of strain MS_2_ increased by 1.7-fold, as compared to that of WT (Fig. 2H). To further assess the effect of accumulated cells on biofilm electroactivity, we compared the electron accepted capacity (EAC) and the electron donated capacity (EDC) for strains MS_1_, MS_2_, MS_12_, and WT. As compared with WT, the EAC and the EDC of MS_2_ were increased by 131% and 61%, respectively, which showed enhanced EET capability and higher electrocatalytic activity (Fig. 2I). Similar results were also obtained with strains MS_1_ and MS_12_. Collectively, our results suggested that increase of cell surface hydrophobicity strengthened interaction between cells and electrode surfaces, which increased the cell coverage on electrode surfaces, promoted biofilm formation, and consequently enhanced EET rate.

### Engineering cell–cell interaction in the adhesion stage

**Fig. 3.**
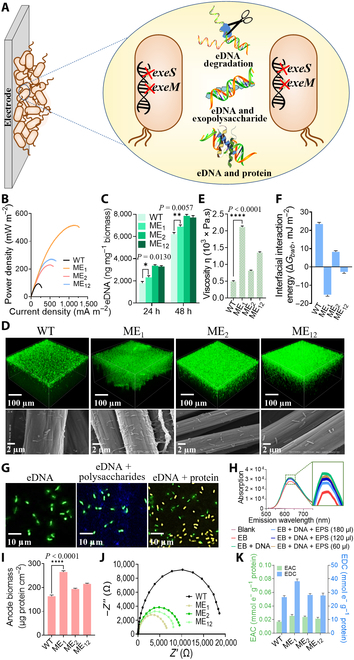
Engineering cell–cell interaction to promote cell–cell adhesion in the adhesion stage (II) of the biofilm formation. (A) Two extracellular endonucleases genes (*exeS* and *exeM*) were knocked out individually and in combination in *S. oneidensis* MR-1 to increase eDNA content in biofilm, leading to the constructs of 3 recombinant strains: ME_1_, ME_2_, and ME_12_. (B) Output power density curves in MFCs of the WT and recombinant strains. (C) eDNA contents of the anode biofilms in MFCs. (D) CLSM (top) and SEM (bottom) images of anode biofilms in MFCs equipped with the WT and recombinant strains. (E) Viscoelastic properties of the biofilms of the WT and recombinant strains. (F) Interfacial interaction energy Δ*G_bwb_* to determine the surface physicochemical parameters of the strains. (G) Co-localization of extracellular polysaccharide (blue), extracellular protein (yellow), and eDNA (green) by CLSM. (H) Fluorescence emission spectra of the EB–DNA complexes at different EPS concentrations. (I) Anode biomass of biofilm formed by recombinant strains in MFCs. (J) Nyquist plots of anodes EIS spectra with *S. oneidensis* recombinant strains in MFCs. (K) EAC and EDC of the recombinant strains in MFCs. Data are presented by 3 independent biological replicates as means ± SD.

eDNA stabilizes cell–cell interaction and structural integrity of biofilm matrix [58*–*61]. In addition to the structural role in biofilm, eDNA was also demonstrated to be a potential nutrient, antimicrobial agent, and reservoir for gene transfer [58,62,63]. Under natural conditions, microbial cells can release nucleases to utilize nutrient [64], control biofilm dispersion, defend themselves from extracellular traps [65], and prevent biofilm formation of other pathogens [66]. However, extracellular endonucleases secreted by cells degrade the eDNA and slow the rate of biofilm formation [43,58,62,63,67]. To increase the level of eDNA in biofilms, we constructed ME_1_, ME_2_, and ME_12_ strains in which the extracellular endonuclease genes *exeS*, *exeM*, and *exeS/exeM* were deleted, respectively (Fig. 3A) [63]. ME_1_, ME_2_, and ME_12_ all exhibited more eDNA in biofilms and generated higher output power density (i.e., 515 ± 19.4, 229 ± 12.7, and 271 ± 11.2 mW m^−2^, respectively) than that of the WT (92 ± 6.5 mW m^−2^) (Fig. 3B and C). As previous reported, many electron shuttling small molecules, such as pyocyanin (PYO), exhibit eDNA binding capacity to be retained in biofilm, thus promoting rapid electron transfer [28,68]. Given that electron transfer occurs rapidly, while loss of electron shuttling small molecules retained by eDNA to the environment is slow, eDNA promotes efficient electron transfer in biofilm [28]. Thus, the increased levels of eDNA in biofilms substantially enhance the EET rate of microbial biofilms to electrode surfaces. CLSM and SEM analyses revealed that the biofilm thickness and coverage on electrode surfaces decreased in the order of ME_1_ (155 μm) > ME_12_ (135 μm) > ME_2_ (125 μm) > WT (120 μm) (Fig. 3D). These results were consistent with colony morphology observations (Fig. S3).

We further found that the viscosity of the biofilm of ME_1_, ME_2_, and ME_12_ was significantly enhanced by 4.36-, 1.67-, and 2.77-fold over WT, respectively (Fig. 3E), verifying that improving cell–cell interaction forces by accumulating eDNA in the adhesion stage indeed facilitated biofilm formation of *S. oneidensis*. To analyze the effect of the thermodynamic properties of eDNA accumulation on cell−cell interaction forces, we further calculated and evaluated the interfacial interaction energy (*ΔG_bwb_*; Eq. 5) of ME_1_ [69]. The negative surface free energy of ME_1_ (−15.18 ± 0.87 mJ m^−2^) and ME_12_ (−2.71 ± 0.79 mJ m^−2^) indicated that the cell–cell interaction of ME_1_ and ME_12_ was stronger and more conducive to cell aggregation than that of ME_2_ (8.23 ± 0.62 mJ m^−2^) and WT (23.49 ± 0.90 mJ m^−2^) (Fig. 3F, Figs. S4 and S5, and Table S3). CLSM co-localization images and DNA-binding assay demonstrated that eDNA of ME_1_ exhibited a stronger bind with polysaccharides and proteins in extracellular polymeric substances (EPS) than that of WT, thus increasing the cell–cell interaction forces and promoting biofilm formation (Fig. 3G and H). Consequently, the biomass attached on the anode surface of ME_1_, ME_2_, and ME_12_ increased by 1.62-, 1.19-, and 1.32-fold, as compared to that of WT, respectively (Fig. 3I). Coincidentally, the internal resistance of ME_1_, ME_2_, and ME_12_ reduced to 26%, 51%, and 43% of WT, respectively (Fig. 3J). With the decrease in internal resistance of the electroactive biofilm, the EAC and EDC of strain ME_1_ increased by 50% and 44%, as compared to that of WT, respectively (Fig. 3K and Eqs. 6 and 7), which showed enhanced bidirectional electron transfer capability and electrocatalytic activity. Similar results were also obtained with strains ME_2_ and ME_12_. Collectively, our results demonstrate that the accumulation of eDNA in the biofilm of *S. oneidensis* improves biofilm formation via increasing the energy and viscosity of microbial cell–cell interaction energy while facilitating the electron transfer in biofilm. This enhances the EET rate of microbial biofilms to the electrode surfaces.

### Increasing the vertical extension of biofilm in the growth stage

**Fig. 4.**
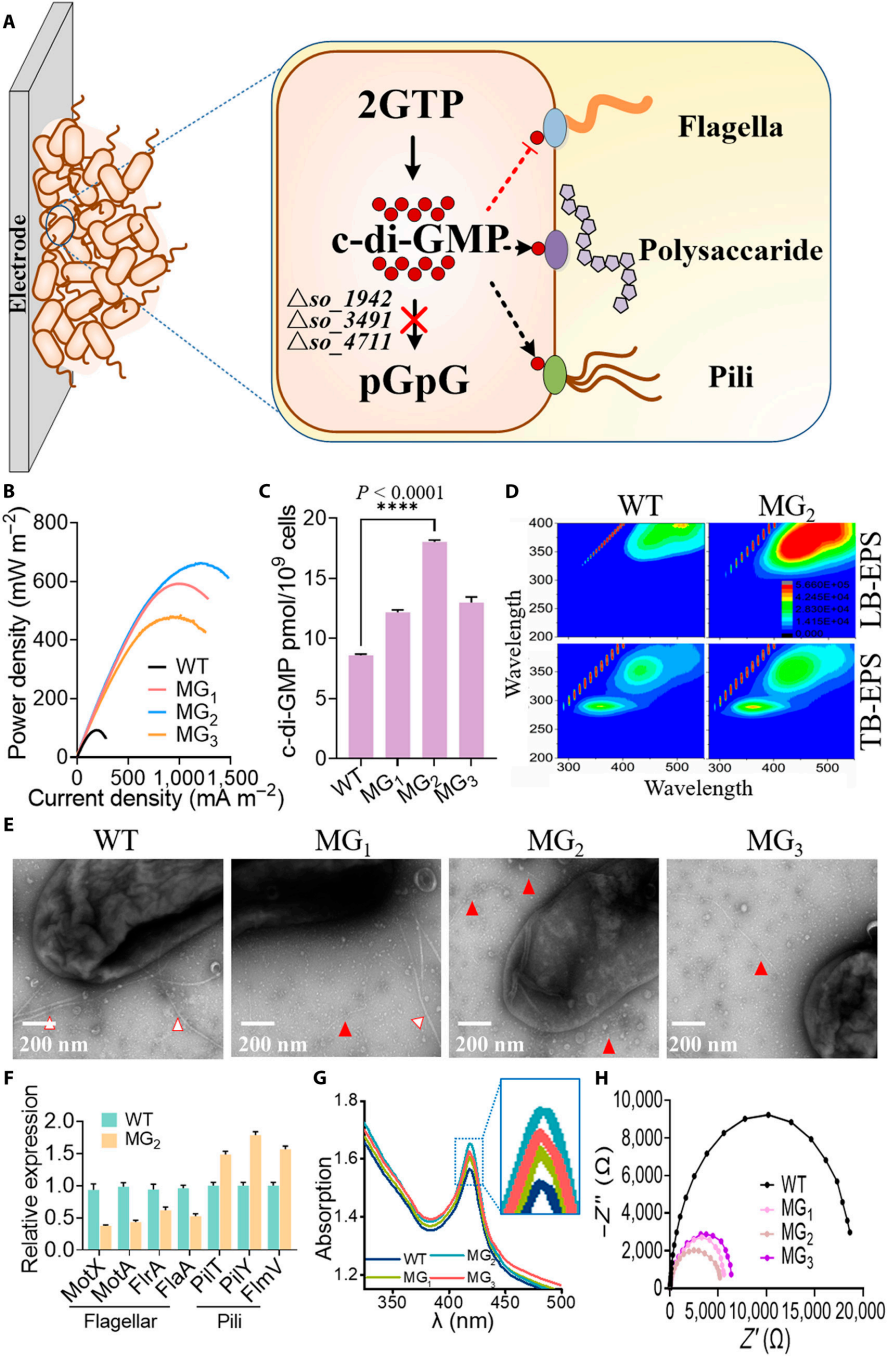
Engineering vertical extension of biofilm in the growth stage (III) of biofilm formation. (A) Three recombinant strains MG_1_, MG_2_, and MG_3_, with disruption of 3 c-di-GMP hydrolase genes (*so1942*, *so3491*, and *so4711*) individually and in combination in *S. oneidensis* MR-1 to reinforce the 3D structure of biofilm. (B) Output power density curves of the strains. (C) c-di-GMP contents of the anode biofilms in MFCs. (D) Three-dimensional excitation–emission matrix fluorescence spectroscopy analyses of tight-bound EPS (TB-EPS) and loose-bound EPS (LB-EPS) of anode biofilms with the *S. oneidensis* recombinant strains. (E) TEM of the recombinant strains. The white triangles point to flagellum, and the red triangles point to pilis. (F) The qRT-PCR analyses of genes related to the synthesis of flagella and pili from the WT and recombinant strains in MFCs. (G) UV-visible spectroscopy analyses of *c*-Cyts in the strains. (H) Nyquist plots of EIS spectra for the anodes with the strains. Data are presented by 3 independent biological replicates as means ± SD.

During the growth stage of biofilm formation, microbial biofilm grows from a monolayer to a 3D structure. c-di-GMP, which is hydrolyzed by phosphodiesterases [70,71], regulates the 3D growth of biofilms. According to previous studies on the comparative genomics of the WT *S. oneidensis* [72], we constructed MG_1_ strain by deletion the gene *so1942* encoding c-di-GMP hydrolases in *S. oneidensis* MR-1 to increase the c-di-GMP level (Fig. 4A). MG_1_ with higher intracellular c-di-GMP level exhibited higher output power density (592 ± 13.1 mW m^−2^) than that of WT (92 ± 6.5 mW m^−2^) (Fig. 4B and C). We wondered whether further increase in the accumulation of c-di-GMP would further increase the output power density. In view of this, we constructed MG_2_ and MG_3_ strains by deletion another 2 phosphodiesterases (namely, *so3491* and *so4711*) sequentially. The output power density decreased in the order of MG_2_ (662 ± 10.8 mW m^−2^) > MG_1_ (592 ± 13.1 mW m^−2^) > MG_3_ (479 ± 11.6 mW m^−2^) > WT (Fig. 4B), while the intracellular c-di-GMP level decreased in the order of MG_2_ > MG_1_ ≈ MG_3_ > WT (Fig. 4C). Thus, increase of intracellular c-di-GMP level improves output power density.

CLSM analyses revealed that the biofilm thickness and furrows depth of MG_1_, MG_2_, and MG_3_ were 1.5-, 1.7-, and 1.6-fold higher than that of WT (120 μm), respectively (Fig. S6A), demonstrating that c-di-GMP accumulation was conducive to the development of a vertical extension structure in biofilm formation. Three-dimensional fluorescence spectrum comparison further verified that the accumulation of c-di-GMP increased the biofilm matrix content, which promoted the development of vertical extension structure (Fig. 4D and Fig. S7). Moreover, we observed that high intracellular c-di-GMP level inhibited flagella formation and promoted pili formation by analyzing piliation at single-cell level using TEM (Fig. 4E). Strains MG_1_, MG_2_, and MG_3_ showed more pili and less flagellar than that of WT. Analysis of flagellar and pili gene expression by quantitative reverse transcription polymerase chain reaction (qRT-PCR) revealed that the relative expression of flagellar genes in strain MG_2_ was decreased from 40% to 60%, while pili genes were significantly increased by 1.5- to 1.8-fold than that of the WT (Fig. 4F). These results were consistent with colony morphology observations (Fig. S6B). Thus, the high c-di-GMP level decreased the strain motility by down-regulating flagella expression and promoted strains twitching on electrode surface by up-regulating pili expression, which consequently enhanced biofilm formation.

Consequently, the biomass attached on the anode surface of MG_1_, MG_2_, and MG_3_ increased by 1.3-, 1.7-, and 1.4-fold, as compared to that of WT, respectively (Fig. S6C). Moreover, the accumulation of c-di-GMP positively regulated the expression of *c*-Cyts involved in the EET conduits of *S. oneidensis* (Fig. 4G). This result was consistent with a previous study, which found that the cells with elevated c-di-GMP were more conductive than that with low c-di-GMP [73]. Coincidentally, the internal resistance of MG_1_, MG_2_, and MG_3_ reduced to 30%, 28%, and 34% of that from WT, respectively (Fig. 4H). The decrease in internal resistance of MG_2_ resulted in an increase of 2.05-fold in EAC and 1.85-fold in EDC, as compared to that of WT (Fig. S6D and Eqs. 6 and 7), suggesting a high electron transfer rate between electroactive cells and electrode surfaces. Similar results were also obtained with strains MG_1_ and MG_3_. Coincidentally, the accumulation of c-di-GMP strengthened the development of vertical extension structure in biofilm formation by increasing biofilm matrix secretion, inhibiting flagella formation, and facilitating pili formation, which consequently led to enhanced EET rate.

### Combinatorial engineering biofilms

**Fig. 5.**
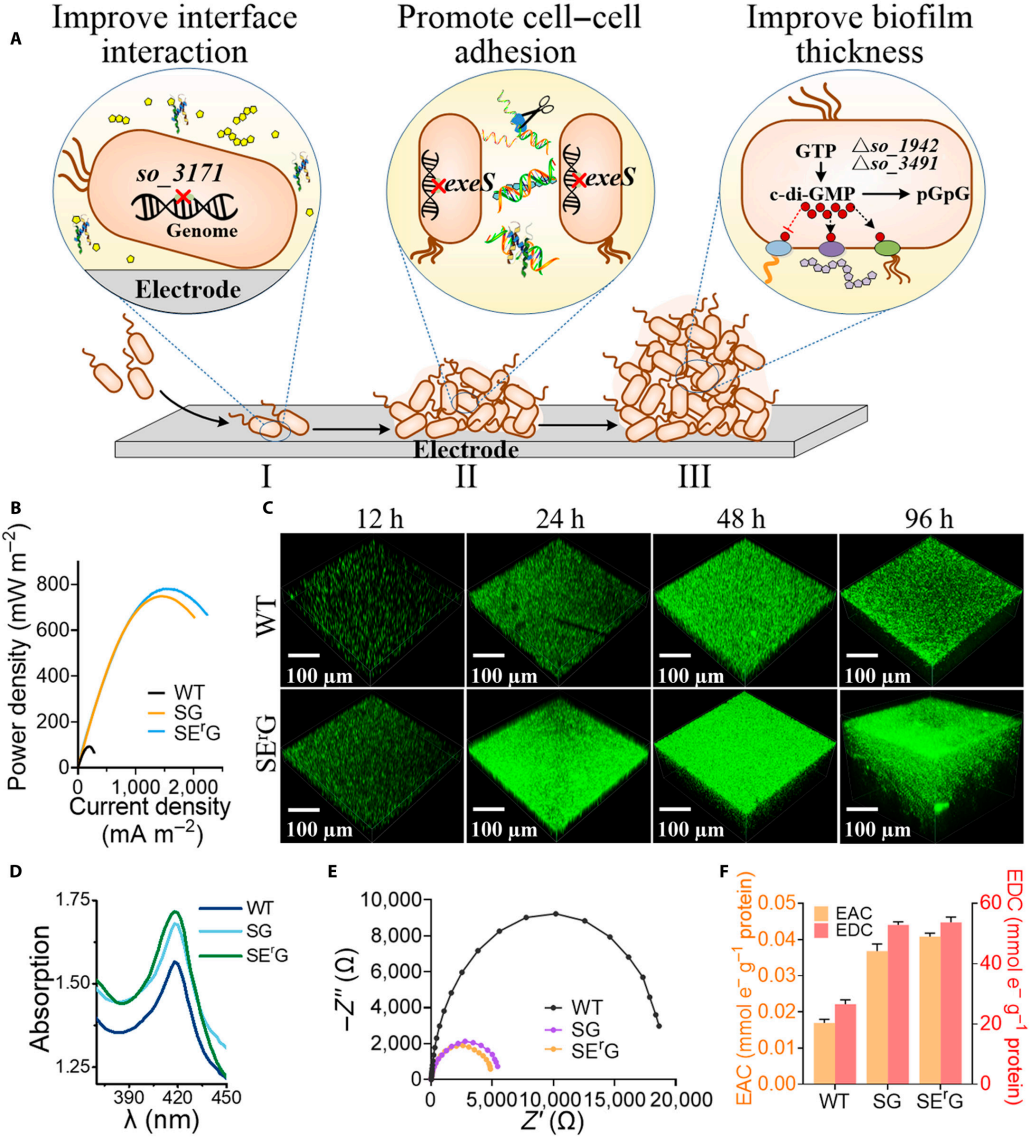
Modular programing of the genes in 3 stages (I to III) of biofilm formation to promote electroactive biofilm formation. (A) *so3171* gene in the cell–electrode initial contact stage (I), *exeS* gene in the cell–cell adhesion stage (II), and *so1942* and *so3491* genes in the biofilm growth stage (III) were selected and programmed to promote electroactive biofilm formation. (B) Output power density curves in MFCs of the WT and strains SG (*so3171*^−^ *so1942*^−^ *so3491*^−^) and SE^r^G (*so3171^−^ so1942^−^ so3491^−^*/pYS), respectively. (C) CLSM images of anode biofilms in the MFCs equipped with the WT and strain SE^r^G. (D) UV-visible spectroscopy analyses of *c*-Cyts in the strains. (E) Nyquist plots of anodes EIS spectra with the strains. (F) EAC and EDC of the strains in MFCs. Data are presented by 3 independent biological replicates as means ± SD.

Given that individual deletion of these genes (*so3171* encodes cell surface polysaccharide biosynthesis enzymes, *exeS* encodes extracellular endonucleases, and genes *so1942* and *so3491* encode c-di-GMP hydrolases) all substantially increased the biofilm formation and conductivity of formed biofilms, we constructed strains SG and SEG in which the genes *so3171/so1942/so3491* and *so3171/exeS/so1942/so3491* were inactivated, respectively (Fig. 5A and Fig. S8). However, SEG displayed impaired ability for growth and electricity production as compared to SG and WT (Fig. S9), in which the knockout of the gene *exeS* would cause detriment to cell growth. To circumvent the difficulty associated with SEG, an alternative strain SE^r^G with antisense RNAs expressed in strain SG was used for down-regulating the expression of the gene *exeS*. The output power density of SE^r^G in MFC was 781 ± 16.5 mW m^−2^, which was higher than that of SG and WT (Fig. 5B).

The biofilm formation of SE^r^G and WT were compared with CLSM. As shown in Fig. 5C, the cell coverage on the electrode surface of SE^r^G was significantly higher than that of WT at 12, 24, and 48 h after biofilm formation. At 96 h after biofilm formation, SE^r^G exhibited much deeper vertical extension and thicker biofilm than WT. In addition, the *c*-Cyts level of strain SE^r^G was increased by 2.2-fold, as compared to that of WT (Fig. 5D). Coincidentally, the internal resistance of strain SE^r^G reduced to 26% of that from WT (Fig. 5E), which resulted in an increase of 2.41-fold in EAC and 2.03-fold in EDC, as compared to that WT (Fig. 5F and Eqs. 6 and 7). Thus, combinatorial engineering biofilms substantially increases biofilm thickness and conductivity.

### Improving conductivity of biofilms by engineering the maturation stage

**Fig. 6.**
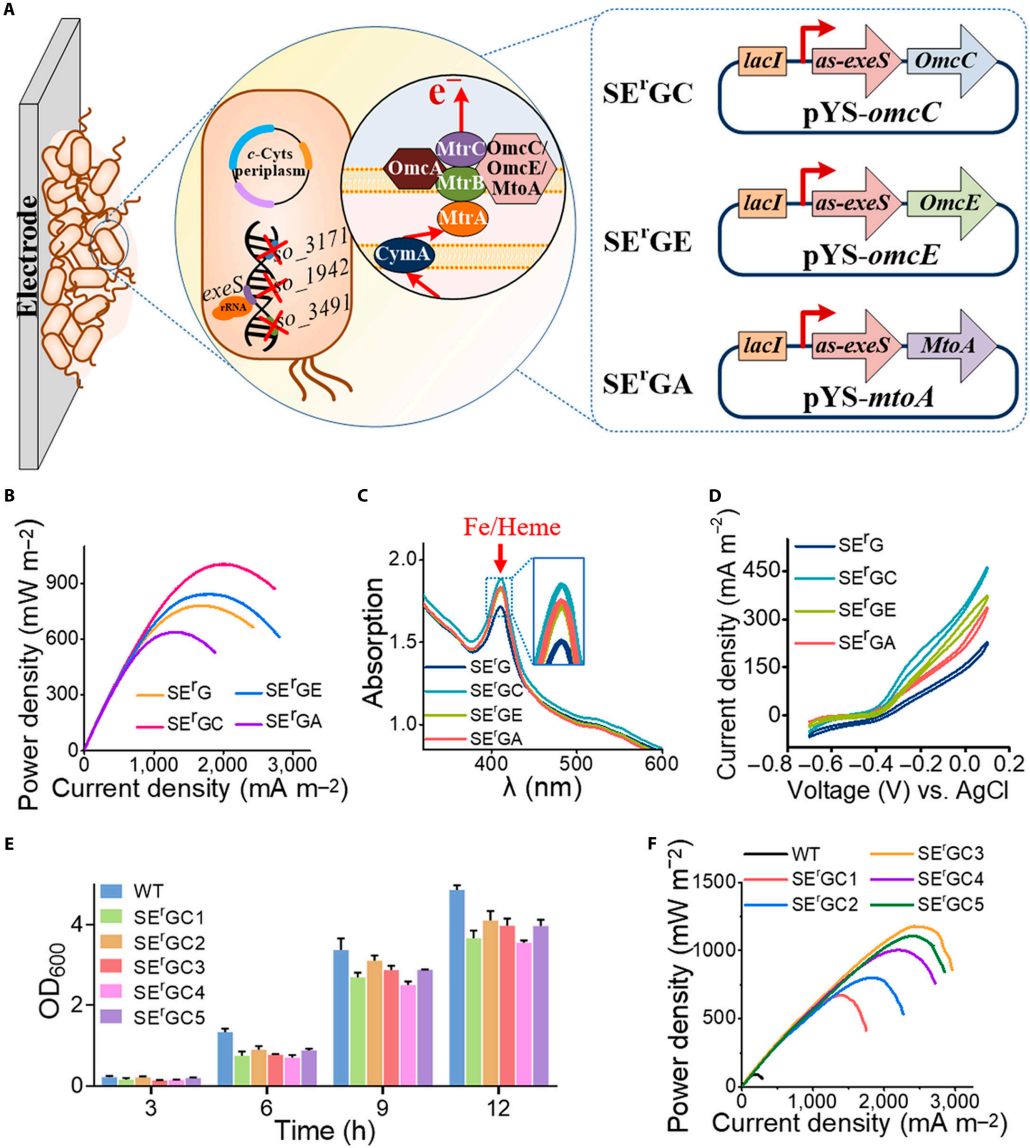
Overexpression of exogenous OM *c*-Cyts to enhance the *c*-Cyts level and EET efficiency in the maturation stage (IV). (A) Three exogenous OM *c*-Cyts (OmcC and OmcE from *G. sulfurreducens* and MtoA from *S. lithotrophicus*) were expressed in strain SE^r^G to construct 3 *S. oneidensis* recombinant strains SE^r^GC, SE^r^GE, and SE^r^GA in order to enhance the *c*-Cyts level and EET rate. (B) Output power density curves in MFCs of the above *S. oneidensis* recombinant strains. (C) UV-visible spectroscopy analyses of *c*-Cyts in the strains. (D) Turnover cyclic voltammetry at a stable scan rate of 1 mV s^−1^. (E) The OD_600_ of the strain SE^r^GC1, SE^r^GC2, SE^r^GC3, SE^r^GC4, and SE^r^GC5 with different RBSs (BBa_B0030, BBa_B0031, BBa_B0032, BBa_B0034, BBa_B0064, and iGEM), respectively. (F) Output power density curves of the strains with different RBSs in MFCs. Data are presented by 3 independent biological replicates as means ± SD.

The cell surface exposed cytochromes of *Shewanella* can act as terminal reductases for external electron-accepting surfaces or intermediary for soluble redox shuttles [74]. However, the inefficient OM *c*-Cyts system limited the EET rate of *S. oneidensis* [26]. To increase the EET rate, 3 exogenous OM *c*-Cyts (OmcC and OmcE of *Geobacter sulfurreducens* and MtoA of *Sideroxydans lithotrophicus*) were expressed in SE^r^G, which resulted in SE^r^GC, SE^r^GE, and SE^r^GA, respectively (Fig. 6A). As shown in Fig. 6B, the output power density SE^r^GC, SE^r^GE, and SE^r^GA were 1,002 ± 19.5 mW m^−2^, 843 ± 17.7 mW m^−2^, and 638 ± 18.3 mW m^−2^, respectively, while that for SE^r^G was 781 ± 16.5 mW m^−2^. Ultraviolet (UV)-visible spectroscopy demonstrated the high expression level of *c*-Cyts in strains SE^r^GC, SE^r^GE, and SE^r^GA (Fig. 6C). We also calculated the cyclic voltammetry curves (Fig. 6D). The peak current density of SE^r^GC was much higher than that of WT, indicating that more redox species were involved in EET of SE^r^GC. On the basis of Laviron equation (Eq. 8), the calculated average density (or activity) of the OM *c*-Cyts on the MFC anode with SE^r^GC was 137.61 ± 5.5 μmol m^−2^ (Fig. 6D), which was 3.1-fold higher that of WT (44.35 ± 3.7 μmol m^−2^). Thus, overexpression of OM *c*-Cyts genes *omcC* resulted in more OM *c*-Cyts for EET, which may contribute to the improved MFC performance and enhanced direct contact-based catalytic current. However, OM *c*-Cyts are membrane protein, which would be toxic to the host by strongly heterologous overexpression [13]. As shown in Fig. 6E, heterologous overexpression of OmcC resulted in a lower optical density at 600 nm (OD_600_) of SE^r^GC than that of WT (Fig. 6E). Thus, we further optimized the ribosome binding site (RBS) sequence to control the translational level of *omcC* in *S. oneidensis*. As shown in Fig. 6F, we identified a weak RBS32 (BBa_B0032, iGEM). Change of original RBS with RBS32 resulted in strain SE^r^GC3. The maximum output power density of SE^r^GC3 was 1,176.2 ± 20.4 mW m^−2^, which was ~12.8-fold higher than that of WT. Most importantly, SE^r^GC3 grew as normally as WT (Fig. 6E). These results clearly demonstrate that the optimized expression of the *omcC* substantially improves the EET rate to electrodes.

**Fig. 7.**
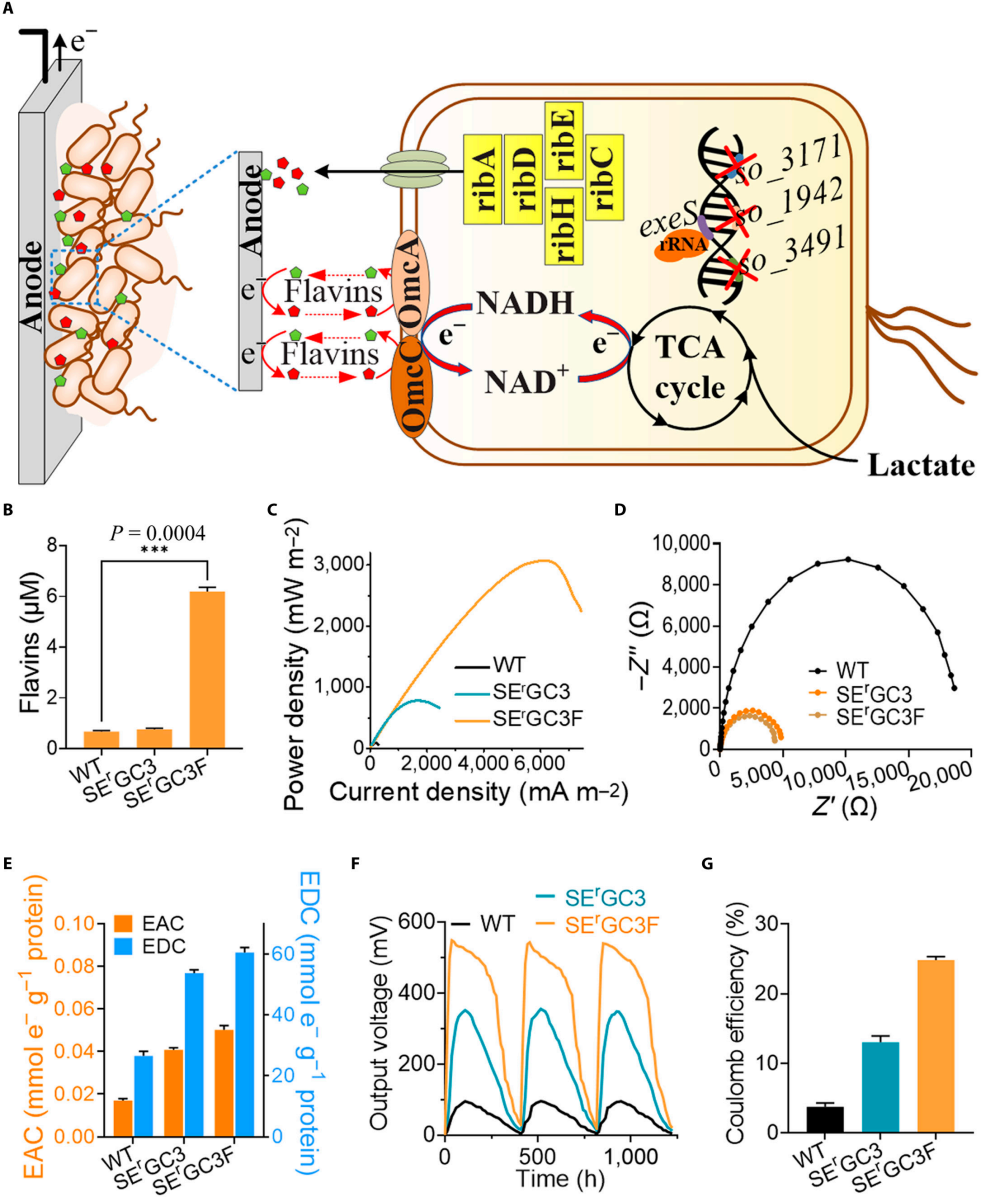
Engineering the conductivity of the naturally occurring biofilms in the maturation stage (IV). (A) The *B. subtilis* flavin biosynthesis gene cluster *ribADEHC* was assembled into strain SE^r^GC3, resulting in strain SE^r^GC3F, in order to facilitate the biosynthesis of flavins and EET efficiency. (B) Quantification of flavins produced by strains WT, SE^r^GC3, and SE^r^GC3F. (C) Output power density curves in MFCs of the above recombinant strains. (D) Nyquist plots of anodes EIS spectra with the above strains. (E) EAC and EDC of the strains above in MFCs. (F) Multiple-cycle voltage output of the WT (black line), strain SE^r^GC3 (blue line), and strain SE^r^GC3F (yellow line) in MFCs. (G) Coulomb efficiency of the WT (black line), strain SE^r^GC3 (blue line), and strain SE^r^GC3F (yellow line) in MFCs. Data are presented by 3 independent biological replicates as means ± SD.

Endogenous electron shuttles flavin mononucleotide (FMN) and RF facilitate EET of *S. oneidensis* [8,74]. To increase the level of flavins in *S. oneidensis*, the synthetic flavin biosynthesis gene cluster *ribADEHC* from *Bacillus subtilis* was incorporated into SE^r^GC3, resulting in the SE^r^GC3F (Fig. 7A). The flavins yield of strain SE^r^GC3F was ~10-fold higher than that of SE^r^GC3 and WT (Fig. 7B). Meanwhile, the maximum output power density of SE^r^GC3F was 3,073.8 ± 42.1 mW m^-2^, which was 2.6- and 33.4-fold higher than that of SE^r^GC3 and WT, respectively (Fig. 7C). The internal resistance of SE^r^GC3F reduced to 23% of that of WT (Fig. 7D). The decreased internal resistance resulted in an increase of 2.96-fold in EAC and 2.28-fold in EDC, as compared to that of WT (Fig. 7E and Eqs. 6 and 7). Thus, the improvement of output power density is related to the accumulation of flavin electron shuttles, which is consistent with the Fick’s law (Eq. 9). The output power density of SE^r^GC3F was also higher than that of SE^r^GC3 (Figs. 6B and 7C), which suggested that EET was substantially improved by combinatorial synthesis of cytochromes and electronic shuttles.

As shown in Fig. 7F, the output voltage of MFCs with a multicycle operation revealed that SE^r^GC3F exhibited stable power generation with a maximum voltage of 548.3 ± 10.4 mV, which was much higher than that of the WT (95.3 ± 3.2 mV). The Coulomb efficiency increased by 6.7-fold from 3.7% (WT) to 24.8% (SE^r^GC3F). These results suggest that the biofilms SE^r^GC3F transfer more electrons to electrodes than that of WT (Fig. 7G and Eq. 1).

### Constructing 3D artificial biofilm in maturity dispersion stage

**Fig. 8.**
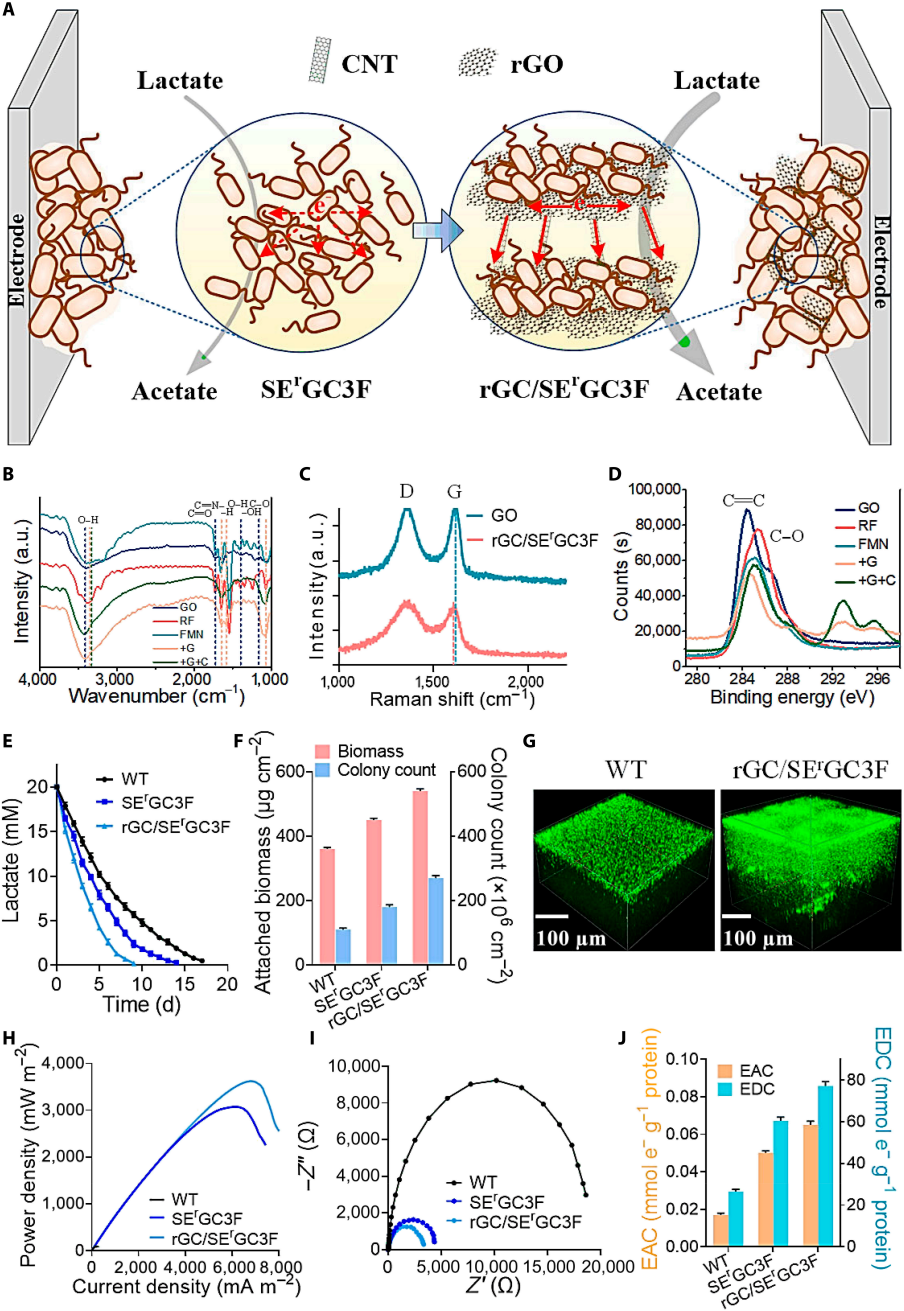
Constructing 3D self-assembled artificial biofilm in maturity dispersion stage (V). (A) Graphene oxide (GO) was employed to construct an artificial 3D self-assembled biofilm in view of the excellent conductive activity and robust spatial distribution controllability. (B) FTIR spectra analyses of GO, RF, FMN, and rGC/SE^r^GC3F artificial biofilm and their tablets. (C) Raman spectra analyses of the GO and the artificial biofilm of rGC/SE^r^GC3F. (D) The C1s XPS spectra analyses of the GO, RF, FMN, and rGC/SE^r^GC3F artificial biofilm. (E) Lactate consumption in the MFCs inoculated with strains WT, SE^r^GC3F, and rGC/SE^r^GC3F, respectively. (F) Biomass and living cells appeared in the biofilm were measured for artificial biofilm. (G) CLSM images of anode biofilms in the MFCs equipped with the WT and rGC/SE^r^GC3F. (H) Output power density curves in MFCs of the above strains. (I) Nyquist plots of anodes EIS spectra with the above strains. (J) EAC and EDC of the strains above in MFCs. “+G” stands for “rG/SE^r^GC3F artificial biofilm,” and “+G+C” stands for “rGC/SE^r^GC3F artificial biofilm.” Data are presented by 3 independent biological replicates as means ± SD.

To further improve the Coulomb efficiency, we employed graphene oxide (GO) to construct an artificial 3D self-assembled artificial biofilm (Fig. 8A). In this artificial biofilm, the bacterial cells were captured by the GO nanosheets by a “fishing” process, where the GO nanosheets acted as nets to catch the bacterial cells. The GO nanosheets were then reduced to rGO by SE^r^GC3F, which self-assembled to form a 3D macroporous conductive network (rG/SE^r^GC3F artificial biofilm). The aggregations were, however, observed in the artificial biofilm of rG/SE^r^GC3F (Fig. S10). These irreversible agglomerates might be formed by π–π stacking between the rG/SE^r^GC3F sheets during the reduction process, which resulted in substantial reduction of surface area [75]. To circumvent this difficulty, we added CNTs to prevent the SE^r^GC3F-G sheets from aggregation. Consequently, artificial biofilm rGC/SE^r^GC3F with 3D conductivity and hierarchical porous was formed. The artificial biofilm rGC/SE^r^GC3F was analyzed by Raman spectra, Fourier transform infrared spectroscopy (FTIR), and x-ray photoelectron spectroscopy (XPS). The Raman and FTIR results showed a structural change upon the reduction of GO by SE^r^GC3F and the attachment of flavins onto rGO (Fig. 8B and C). XPS results confirmed the formation of rGO artificial biofilm (Fig. 8D).

The lactate metabolic rate of artificial biofilm rGC/SE^r^GC3F was 2.2 ± 0.01 mM d^−1^, which was 2-fold higher than that of WT (1.1 ± 0.01 mM d^−1^) (Fig. 8E). The increased mass transfer efficiency promoted the cell metabolism and growth. As a result, the measured biomass for artificial biofilm SE^r^GC3F-GC was 1.2- and 1.5-fold than that of artificial biofilm SE^r^GC3F and the WT, respectively (Fig. 8F). The living cells in the biofilm increased by 2.5-fold, as compared to that of the WT (Fig. 8F). Therefore, the construction of artificial biofilm rGC/SE^r^GC3F reduced the spatial and chemical heterogeneity and promoted the formation of biofilm. As revealed by CLSM images, the thickness of artificial biofilm rGC/SE^r^GC3F was 305 μm, which was 2.5-fold higher than that of WT (120 μm) (Fig. 8G). In addition to enhancing EET through increased mass transfer efficiency near the electrode for adhering more bacteria and assisting the formation of dense biofilm, the rGO–biofilm hybrid can also generate and enrich local electron shuttles at a higher concentration around the anode and decreased the diffusion distance of electron shuttles. The maximum output power density of artificial biofilm rGC/SE^r^GC3F was 3620 ± 56.2 mW m^−2^, 39.3-fold higher than that of WT (92 ± 6.5 mW m^−2^) (Fig. 8H), which is, to the best our knowledge, the highest output power density that has ever been reported in engineered electroactive biofilm of *Shewanella* (Table S1). Additionally, the internal resistance of artificial biofilm rGC/SE^r^GC3F reduced to 18% of that of WT (Fig. 8I). The decreased internal resistance of electroactive biofilm resulted in increase of 3.8-fold EAC and 2.9-fold EDC, as compared to that of WT (Fig. 8J and Eqs. 6 and 7). The decreased internal resistance and the increased EAC and EDC values reflect the high electron collection of artificial biofilm rGC/SE^r^GC3F and the formation of a stable electron transfer network. Therefore, the artificial biofilm rGC/SE^r^GC3F exhibited stable power generation with a maximum voltage of 658.1 ± 9.6 mV and a maximum Coulomb efficiency of 30.7%, which were 6.9- and 8.3-fold higher than that of WT, respectively (Fig. S11). Together, these construct artificial conductive channels in rGC/SE^r^GC3F 3D self-assembled artificial biofilm improve electron transfer in biofilm and electron transfer from biofilms to the anode surfaces and diminish the dispersion stage of biofilm.

## Conclusion

We rationally designed an artificially electroactive biofilm with high electrical conductivity. This was carried out via systematic engineering biofilms to promote biofilm forming ability and enhance biofilm conductivity from the full-cycle perspective. It included improvement of cell membrane hydrophobicity by improving the interactions between microbial cells and electrode surfaces in the initial contact stage. Accumulation of eDNA was then increased to improve cell–cell interaction forces and structural integrity of biofilm in the adhesion stage. The c-di-GMP level was also increased to reinforce the vertical extension and 3D structure formation of biofilm in the growth stage. By strengthening biofilm formation, the output power density was improved from 92 ± 6.5 mW m^−2^ (WT) to 781 ± 16.5 mW m^−2^. Synthesis of OM *c*-Cyts and RF was strengthened to enhance EET rate of biofilm in the stable maturity stage, leading to an output power density of 3,073.8 ± 42.1 mW m^−2^ (33.4-fold higher than the WT). On the basis of this, an engineered rGC/SE^r^GC3F 3D self-assembled artificial biofilm was further constructed to increase the electron collection capacity and reduce internal resistance of artificial electroactive biofilm in maturity dispersion stage, resulting the highest record of the output power density 3.62 ± 0.06 W m^−2^ (39.3-fold higher than the WT), which is, to the best our knowledge, the highest record in engineered *S. oneidensis* biofilms ever reported. Such artificial electroactive biofilm would lay a foundation for practical applications of electrocatalytic systems in the fields of energy, chemical industry, environments, and bioelectronics.

## Materials and Methods

### In vitro gene synthesis

The sequences of the gene *aggA* of *S. oneidensis*, the gene *rbmA* of *V. cholerae*, the gene *saeP* of *S. aureus*, the genes *omcC* and *omcE* of *G. sulfurreducens*, the gene *mtoA* of *S. lithotrophicus*, and the gene *ribACDEH* of *B. subtilis* were obtained from the National Center for Biotechnology Information database (Table S7) and expressed in *S. oneidensis* with codon optimization as previously reported [7]. The information of *so_1860*, *so_3171*, *exeS*, *exeM*, *so_1942*, *so_3491*, and *so_4711* genes of *S. oneidensis* were extracted from the National Center for Biotechnology Information database. The designed gene sequences were synthesized by GENEWIZ in vitro (Suzhou, China).

### Plasmid construction and bacterial culture

The strains used in this study are listed in Table. All plasmid constructions were performed in *Escherichia coli* WM3064 (auxotroph), with 100 μg ml^−1^ of 2,6-diaminopimelic acid and 50 μg ml^−1^ of kanamycin added when needed. The obtained genes were assembled into pYYDT as previously constructed in our laboratory [76]. The constructed plasmid was first transformed into *E. coli* WM3064, a plasmid donor strain. Then, conjugation of plasmid donor strain and *S. oneidensis* was employed to obtain engineered *S. oneidensis*. The gene mutant strains of *S. oneidensis* were constructed using the *att*-based fusion PCR method [77,78].

**Table.** Strains used in this study.

**Strains**	**Description**	**Source**
MR-1	*Shewanella oneidensis* MR-1 wild-type strain	Our lab
MS_1_	MR-1 Δ*1860*	This study
MS_2_	MR-1 Δ*3171*	This study
MS_12_	MR-1 Δ*1860*Δ*3171*	This study
MP_1_	MR-1 carrying pYYDT-*aggA*	This study
MP_2_	MR-1 carrying pYYDT-*rbmA*	This study
MP_3_	MR-1 carrying pYYDT-*saeP*	This study
ME_1_	MR-1 Δ*exeS*	This study
ME_2_	MR-1 Δ*exeM*	This study
ME_12_	MR-1 Δ*exeS*Δ*exeM*	This study
MG_1_	MR-1 Δ*1942*	This study
MG_2_	MR-1 Δ*1942*Δ*3491*	This study
MG_3_	MR-1 Δ*1942*Δ*3491*Δ*4711*	This study
SG	MR-1 Δ*1942*Δ*3491*Δ*3171*	This study
SEG	MR-1 Δ*1942*Δ*3491*Δ*3171*Δ*exeS*	This study
SE^r^G	SG carrying pYYDT-*as-exeS* (pYS)	This study
SE^r^GC	SG carrying pYYDT-*as-exeS-omcC*	This study
SE^r^GE	SG carrying pYYDT-*as-exeS-omcE*	This study
SE^r^GA	SG carrying pYYDT-*as-exeS-mtoA*	This study
SE^r^GC1	SG carrying pYYDT-*as-exeS-RBS30-omcC*	This study
SE^r^GC2	SG carrying pYYDT-*as-exeS-RBS31-omcC*	This study
SE^r^GC3	SG carrying pYYDT-*as-exeS-RBS32-omcC*	This study
SE^r^GC4	SG carrying pYYDT-*as-exeS-RBS34-omcC*	This study
SE^r^GC5	SG carrying pYYDT-*as-exeS-RBS64-omcC*	This study
SE^r^GF	SG carrying pYYDT-*as-exeS-ribACDEH*	This study
SE^r^GC3F	SG carrying pYYDT-*as-exeS-ribACDEH-RBS32-omcC*	This study
rG/SE^r^GC3F	SE^r^GC3F with rGO	This study
rGC/SE^r^GC3F	SE^r^GC3F with rGO and CNTs	This study

### Bio-electrochemical systems (BES) setup

To evaluate the capacity of EET, the overnight culture suspension of *S. oneidensis* was inoculated into fresh Luria–Bertani (LB) broth at 1% inoculum and incubated at 30 °C, 200 rpm, until the OD_600_ reached ∼2.0. The harvested cells were washed thrice with fresh M9 buffer and subsequently resuspended in 140 ml of electrolyte (M9 buffer was supplemented with 5% LB, 20 mM lactate, 0.5 mM isopropyl-β-d-thiogalactopyranoside, and 50 μg ml^−1^ of kanamycin). Dual-chamber MFCs (140-ml working volume) were separated using Nafion 117 membranes (DuPont Inc., USA). Carbon cotton was used as the electrodes for the anode (1.0 cm × 1.0 cm) and carbon cloth for cathode (2.5 cm × 3 cm). The cathodic electrolyte was prepared with 50 mM K_2_HPO_4_, 50 mM KH_2_PO_4_, and 50 mM K_3_[Fe(CN)_6_]. The voltage was measured across a 2-kΩ external resistor in the external circuit and recorded using a multimeter (DT9205A).

### Artificial biofilm construction and characterization

CNT was adsorbed to the anode by soaking and drying repeatedly. *S. oneidensis* cell suspension was added according to the method in BES setup and dispersed into the modified anodes. Then, the anode chamber was added with GO to 0.2 mg ml^−1^, purged with N_2_ gas, and stirred at 30 °C, 50 rpm to prepare the 3D artificial biofilm. The Raman spectrometer (Horiba, France) with a 632-nm laser source, FTIR spectrometer (MKS6030, MKS), and XPS measurements (ThermoFisher K-Alpha, USA) with a monochromated AlKα radiation were performed after the output voltage of MFC stabilized.

### Electrochemical analysis

Cyclic voltammetry analysis with a scan rate of 1 mV s^−1^ and electrochemical impedance spectroscopy (EIS) analysis were performed on a CHI 1000C multichannel potentiostat (CH Instrument, Shanghai, China) in a 3-electrode configuration, and an Ag/AgCl was used as reference electrode. EIS was conducted at a set potential equal to the OCP, with a sinusoidal perturbation of 5-mV amplitude, over a frequency range of 10 mHz to 100 kHz.

The Coulomb efficiency (*C_E_*) is defined as a ratio of the Coulombs recovered by the actual current to maximum possible Coulombs of current produced by all substrate consumption [79] as Eq. 1:

$$C_{E}=\frac{\text{Coulombs recovered}}{\text{Total coulombs in substrate}}=\frac{M_{S}\int_{0}^{t_{b}}\text{Idt}}{Fb_{\text{ES}}V_{\text{An}}△c}=\frac{M_{S}It_{b}}{Fb_{\text{ES}}V_{\text{An}}△c}$$ (1)

where *M_s_* is 90.08 g mol^−1^ (lactate molecular weight), *F* is 98,485 C mol^−1^ of electrons (Faraday’s constant), *V* is 0.14 l (the liquid volume of anode compartment), *b_ES_* is 4 (the stoichiometric number of moles of electrons produced per mole of lactate), *t_b_* (s) is the period of a batch cycle, *I* (A) is the current, *Δc* (g l^−1^) is the change in substrate concentration over time *t_b_*.

### Confocal imaging of anode biofilm

Anode biofilms were harvested and washed thrice with phosphate-buffered saline (PBS) (pH 6.8). Then, confocal images of anode biofilm were observed using LIVE/DEAD BacLight Bacterial Viability Kit (Invitrogen, U.S.A.) and CLSM (Nikon A1R+) and were analyzed by the Nikon A1R+ software with laser wavelengths of 488 and 561 nm. For the observation of EPS, fluorescein isothiocyanate and concanavalin were used for staining the extracellular protein and extracellular polysaccharide, respectively.

### SEM imaging of the anode biofilm

The anode carbon cotton was harvested from operating MFCs and fixed in 2.5% glutaraldehyde for 12 h. The anode carbon cotton was subsequently dehydrated in different concentration gradients of ethanol solution (30%, 50%, 70%, 80%, and 90%) and vacuum-dried overnight. The imaging of the anode biofilm was characterized by SEM (S-4800, Hitachi) with 3-kV acceleration voltage. Samples were divided into small pieces and coated with Au before the SEM observing.

### Measurement of anode biomass

To determine the protein content of the anode biofilm, the anode carbon cotton was vortexed in 3-ml PBS for 2 min and then incubated 20 min at 96 °C to lyse cells. Bicinchoninic acid (BCA) protein assay kit (Solarbio, China) was used for the test of extracts according to the manufacturer’s instructions with 1 mg ml^−1^ of bovine serum albumin as standard.

### Congo red assay

*Shewanella* cells were inoculated onto Congo red plates containing 0.01% (wt vol^−1^) Congo red and 1.5% (wt vol^−1^) agar. After growth at 30 °C for 3 d, clearing zones were measured around colonies.

### Ruthenium red staining

*Shewanella* cells were harvested from the anode biofilm for preparing ruthenium red-stained samples and were fixed and stained for 5 h at 4 °C as previously reported [72]. Then, the cells were washed with 0.1 M cacodylate buffer (pH 6.5) before a buffer containing 1% osmium tetroxide and 0.05% ruthenium red were being used to postfix at 4 °C for 2 h. The cells were embedded in 1.5% agarose after rinsing with the cacodylate buffer, and a graded ethanol series was used for dehydration. The cells without ruthenium red staining were performed as control. The dehydrated blocks of both experimental group and control group were embedded in Spurr resin. Before staining with lead citrate and uranyl acetate, ultrathin sections were prepared using an ultramicrotome and then fixed on copper meshes. The images of cell were obtained by TEM at 75 kV (H-7000, Hitachi).

### Hydrophobicity assay

*Shewanella* cells were suspended in 0.15 M NaCl solution until the OD_600_ reached ∼0.3. Then, the cell suspension was mixed with 0.4-ml hexadecane and vortexed for 60 s. After standing for 15 min at room temperature to allow complete separation of the 2 phases, 1-ml sample of aqueous phase was obtained for measuring the OD_600_. Affinity, defined as a percentage of cells from aqueous phase to organic phase, is written as Eq. 2:

$$\text{Affinity}\;(\%)=100\times [1-(A/A_{0})]$$ (2)

where *A*_0_ and *A* are the OD_600_ values of the sample of aqueous phase before and after mixing with organic phase (here, hexadecane), respectively. Each sample was tested 3 times.

### Contact angle measurement

Hydrophobicity of the engineered electroactive biofilm was detected by contact angle measurement. Isolated biofilm samples were first prepared by carefully cutting LB solid plate to ensure an undamaged surface of biofilms and then placed onto a glass slide horizontally. The contact angle measurement was performed with 3 liquids (water, diiodomethane, and formamide) (2 μl) at room temperature using an automated goniometer (JC2000DM, Powereach, Shanghai, China) following the sessile drop method (Table S4). Each sample was tested 3 times.

### Hydrophobicity of cell surface

The hydrophobicity of bacteria cell surface can be measured by the surface tension components [69]. According to the Young’s equation, the surface tension is determined by the contact angle. For bacteria, the surface tension is written as Eq. 3:

$$\frac{1}{2}(1+\cos\theta )\gamma_{L}=\sqrt{\gamma_{b}^{\text{LW}}\gamma_{L}^{\text{LW}}}+\sqrt{\gamma_{b}^{+}\gamma_{L}^{-}}+\sqrt{\gamma_{b}^{-}\gamma_{L}^{+}}$$ (3)

where *θ* is the contact angle of the droplet on the bacteria lawn, *γ_L_* is the total surface tension of the liquid, *γ^LW^* is the Lifshitz–van der Waals surface tension component, and *γ*^+^ and *γ^–^* are the electron–acceptor and electron–donor component, respectively. The subscript *L* denotes the liquid used to determine the contact angle; the subscript *b* stands for the bacterial lawn.

The *γ_b_^+^*, *γ_b_^–^*, and *γ_b_^LW^* can be calculated using 3 known surface tension fluids. The total surface tension (*γ_b_*) of the bacteria lawn is written as Eq. 4:

$$\gamma_{b}=\gamma_{b}^{\text{LW}}+2\sqrt{\gamma_{b}^{+}\gamma_{b}^{-}}$$ (4)

On the basis of the surface tension parameters, the interfacial interaction energy (Δ*G_bwb_*) is calculated for the quantitative measurement of cell surface hydrophobicity. A negative value for the free energy (Δ*G_bwb_*) between 2 similar surfaces immersed in water is defined as a hydrophobic surface. The free energy (Δ*G_bwb_*) between bacteria lawn and water is written as Eq. 5:

$$\begin{array}{c} △G_{\text{bwb}}=-2{\left(\gamma_{b}^{\text{LW}}+\gamma_{w}^{\text{LW}}\right)}^{2}\\ -4\left(\sqrt{\gamma_{b}^{+}\gamma_{b}^{-}}+\sqrt{\gamma_{w}^{+}\gamma_{w}^{-}}-\sqrt{\gamma_{b}^{+}\gamma_{w}^{-}}-\sqrt{\gamma_{b}^{-}\gamma_{w}^{+}}\right) \end{array}$$ (5)

where the subscript *w* stands for water.

### Electron exchange capacity analysis

The 3-electrode cell was used for measure, and an Ag/AgCl electrode was used as the reference electrode. The working electrode potential of EAC was set to −0.49 V, and EDC was set to +0.61 V. The EAC and EDC, which are calculated by integrating the reductive and oxidative current peaks, are written as Eqs. 6 and 7:

$$\text{EAC}=\frac{\int \frac{I_{\mathrm{red}}}{F}\text{dt}}{m_{\text{protein}}}$$ (6)

$$\text{EDC}=\frac{\int \frac{I_{\text{ox}}}{F}\text{dt}}{m_{\text{protein}}}$$ (7)

where *I*_red_ (A) is the reductive current, *I*_ox_ (A) is the oxidative current, *F* is 98,485 C mol^−1^ of electrons (Faraday’s constant), and *m*_protein_ (gram of protein) is the mass of anode protein. To ensure baseline separation, each sample was tested 3 times with more than 30 min between the analyses.

### Quantification of eDNA

The supernatant of anode biofilm was harvested after 24 and 48 h of incubation and filter sterilized. The Quant-iT PicoGreen dsDNA Reagent (Invitrogen/Molecular Probes, Darmstadt, Germany) was used to analyze the eDNA in the biofilm supernatant. The quantification of eDNA in the supernatant was determined according to the operation method of the instruction manual.

### Viscosity measurement

The viscosity properties of biofilm were analyzed with a DNR rheometer (DHR-2) at room temperature. The biofilm sample scraped from the LB plate was placed on the 25-mm-diameter cone plate (101-μm gap) with strain amplitude ranging from 0.01% to 10% at a constant frequency of 10 rad s^−1^, and the shear rates ranging from 0.001 to 100 s^−1^.

### Extraction and quantification of c-di-GMP

The extraction and quantification of c-di-GMP were performed as previously described with modifications [73]. Cell pellets in the mid-log phase were harvested at 4 °C and washed for 2 times using 10 mM PBS. Then, the cell pellets were resuspended in 300-μl ammonium acetate (1 mM, ice-cold). The cell suspension was vortex for 15 s at 4 °C and then centrifuged for 10 min at 4 °C; the supernatant was harvested. This operation was repeated at least 3 times, and all of the supernatants were collected together, which were following vacuum-dried overnight. The white particles that appeared after evaporation were the samples containing c-di-GMP and were stored at −80 °C for testing. The c-di-GMP samples were quantified by high-performance liquid chromatography with a C18 column (2.1 × 40 nm, 5 μm). Specifically, the c-di-GMP was isolated through the gradient elution with the 0.2 ml min^−1^ of flow rate, in which the mobile phase were A: 0.77 g l^−1^ of ammonium acetate aqueous solution and B: 0.77 g l^−1^ of ammonium acetate methanol solution (0 to 9 min, 99% A; 9 to 14 min, 85% A; 14 to 19 min, 75% A; 19 to 26 min, 10% A; 26 to 40 min, 99% A), and the UV detection (253 nm) was used for c-di-GMP detection. All experiments were performed in triplicates.

### EPS extraction

The cells of the biofilm electrode were harvested and washed 2 times with 0.9% saline. After centrifuging the washed cell pellets at 4 °C, the cells were further removed via filtering collected supernatants through 0.22-μm membranes (ANPEL Laboratory Technologies, China), and the filtrates were collected as loose-bond EPS samples and stored at −20 °C before use. Washed cell pellets were resuspended in 0.9% saline with 26.9 mM Na_2_-EDTA and stirred at 4 °C for 4 h. Cell pellets were centrifuged (5,000 rpm, 20 min, 4 °C) and resuspended in 0.9% saline. Washed cell pellets were centrifuged (5,000 rpm, 15 min, 4 °C), and the cells were removed via filtering collected supernatants through 0.22-μm membranes. Finally, the obtained filtrates were collected as tight-bond EPS samples and stored at −20 °C before use.

### Excitation–emission matrix fluorescence spectroscopy

A steady-state fluorescence spectrometer (Jobin Yvon Fluorolog 3-21) was used to measure the 3D excitation-emission matrix spectra. The scanning range of emission spectra was 300 to 550 nm at 0.5-nm increments, and the scanning range of excitation spectra was 200 to 400 nm at 10-nm increments. Ten nanometers was set as the slits of excitation and emission with the scanning speed of 1,200 nm min^−1^. The ddH_2_O (double-distilled water) was set as the blank.

### qRT-PCR

Bacterial total RNA of the mid-log phase was isolated using a Bacterial Total RNA Extraction Kit (APEXBIO, China). cDNA was synthesized through the GoScript reverse transcription system (Promega, USA); the target gene expression was quantified through the Sso Advanced SYBR Green Supermix (Bio-Rad, USA). The *gyrB* acts as the reference gene, of which the expression level was used to normalize the expression levels of the target genes. Primers used for the amplification of target gene were displayed in Table S5. The 2^−ΔΔCT^ method was used for data analysis.

### Observation of gliding motility

To observe the gliding motility of bacteria, 10 μl of bacterial cells was inoculated on the LB agar plate for 10 h at 30 °C. The bacterial behavior was recorded for further analysis.

### Quantification of *c*-Cyts

The cells of the biofilm electrode were harvested and resuspended by 10 mM PBS for ultrasonication. The *c*-Cyts in the cell lysate were analyzed and quantified using a UV-visible spectrophotometer (UV-2450, Shimadzu).

### Quantification of RF and lactate

The supernatant of the MFCs was centrifuged (35000 rpm for 20 min) and filtered (0.22 μm). The eluted samples were analyzed for RF and lactate using liquid chromatograph-tandem mass spectrometer (Agilent LCMS-1290−6460) and high-performance liquid chromatography, respectively. For the quantification of RF, a Waters XBridge C8 column (2.1 mm × 100 mm; particle size: 3.5 μm) was used in positive ion mode. Lactate was quantified by an organic acid column (Aminex HPX-87H Column, 300 mm × 7.8 mm, Bio-Rad) with a refractive index detector (Waters, Corp.) at 65 °C. H_2_SO_4_ (5 mM) acted as the mobile phase at a flow rate of 0.6 ml min^−1^.

### Laviron equation

The relationship between the outer-membrane cytochrome density (*Γ*) and the peak current (*I_p_*) and is written as Eq. 8:

$$I_{p}=\frac{n^{2}f^{2}\text{Av}\Gamma }{4\text{RT}}$$ (8)

where *I_p_* represented the peak current, *n* is the electron transfer number (here, *n* = 1), *A* is the anode area, *F* is the Faraday’s constant, *R* is the gas constant, and *T* is the experiment temperature.

### Fick’s law

The transport of soluble electron shuttles is mainly carried out by diffusion following Fick’s law and can be shown to reflect current density as Eq. 9:

$$j=\text{nF}\left(\frac{D_{\text{shuttle}}∆C_{\text{shuttle}}}{∆z}\right)$$ (9)

where *j* is the current density (A m^−2^), *D*_shuttle_ (m^2^ s^−1^) is the diffusion coefficient of the soluble electron shuttle, ∆*z* (m) is the electron transport distance, ∆*C*_shuttle_ (mol m^−3^) is the concentration gradient of oxidized/reduced shuttle, and nF is the convert coefficient from moles to Coulombs.

## Data Availability

All data needed to evaluate the conclusions in the paper are present in the paper and/or the Supplementary Materials.

## Supplementary Materials

Supplementary 1 Figs. S1 to S11 Tables S1 to S7 References [80–89]
