# Supplementary material for: Systematic Full-Cycle Engineering Microbial Biofilms to Boost Electricity Production in Shewanella oneidensis
Source: Research (Wash D C). 2023 Mar 15;6:0081. doi: 10.34133/research.0081 (PMC10017123; doi:10.34133/research.0081)
Supplement: Supplementary 1 — Figs. S1 to S11 Tables S1 to S7 References [80–89] [file research.0081.f1.docx]

**Supplementary Materials for**

**Systematic Full-cycle Engineering Microbial Biofilms to Boost Electricity Production in *Shewanella oneidensis***

Feng Li^1,2,‡^, Rui Tang^1,2,‡^, Baocai Zhang^1,2,‡^, Chunxiao Qiao^1,2^, Huan Yu^1,2^, Qijing Liu^1,2^, Junqi Zhang^1,2^, Liang Shi^3^, and Hao Song^1,2,*^

^1^ Frontiers Science Center for Synthetic Biology (Ministry of Education), and Key Laboratory of Systems Bioengineering, Tianjin University, Tianjin, 300072, China.

^2^ Collaborative Innovation Center of Chemical Science and Engineering (Tianjin), School of Chemical Engineering and Technology, Tianjin University, Tianjin, 300072, China.

^3^ Department of Biological Sciences and Technology, School of Environmental Studies, China University of Geoscience in Wuhan, Wuhan, Hubei 430074, China.

‡ Equal contribution.

* Corresponding author. E-mail: [hsong@tju.edu.cn](mailto:hsong@tju.edu.cn)





**Fig. S1 Surface physicochemical properties of recombinant strains with disruption of encoding cell surface polysaccharide-biosynthesis enzymes genes.** Determination of the cell surface hydrophobicity of recombinant strains under the same condition using the percentages of cells from aqueous phase to hydrophobic organic phase hexadecane. Data are presented by three independent biological replicates as mean ± SD.


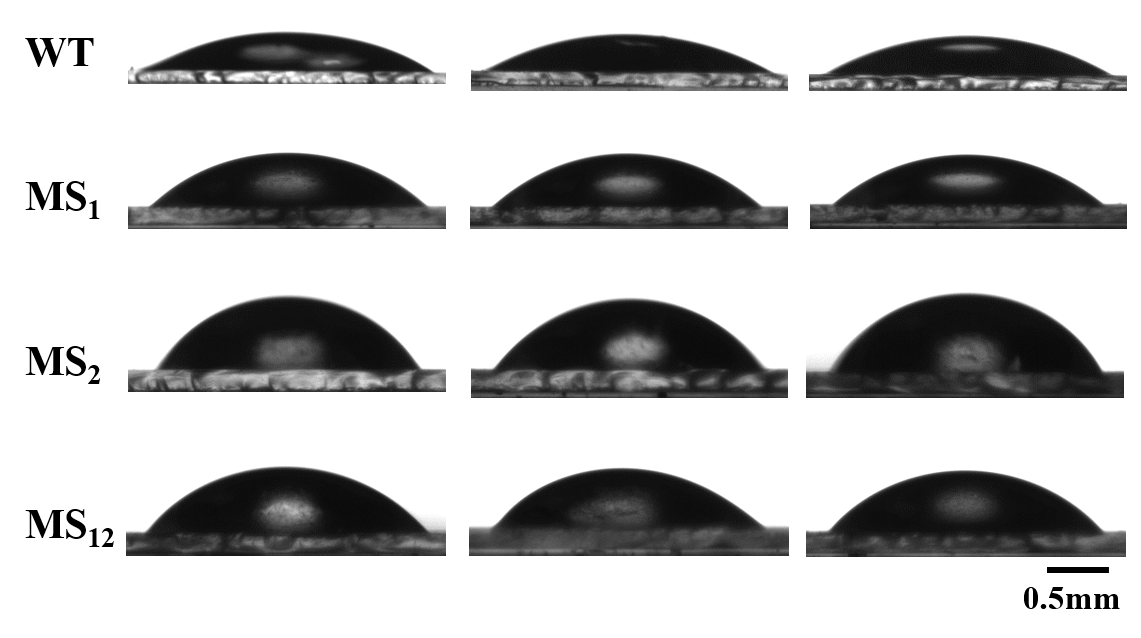


**Fig. S2 Images of contact angles.** Images of contact angles measured by dropping a drop of water on the surface of bacterial cells of different *S. oneidensis* recombinant strains with disruption of *so3171* and *so1860* genes encoding cell surface polysaccharide-biosynthesis enzymes individually and in combination, respectively.


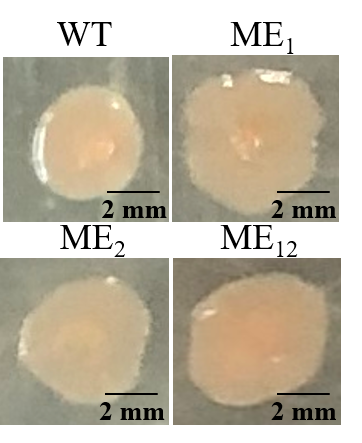


**Fig. S3 Colony morphologies.** Colony morphologies of the recombinant strains ME_1_, ME_2_, and ME_12_.

**
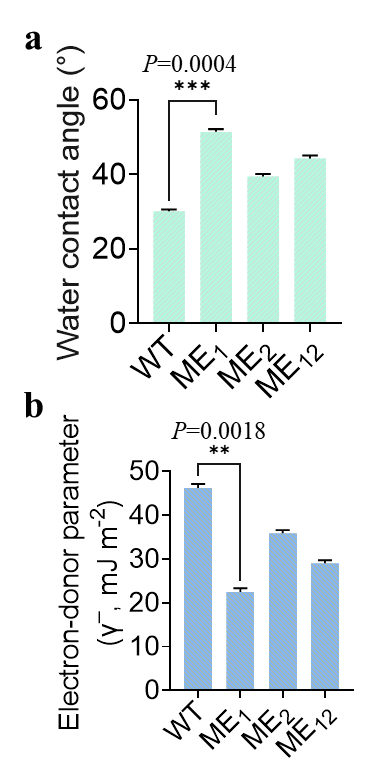
**

**Fig. S4 Surface physicochemical properties of *S. oneidensis* recombinant strains with disruption of encoding extracellular endonucleases genes.** Under the same condition, the surface physicochemical parameters of *S. oneidensis* strains were determined by the water contact angle *θ_W_* **(a)** and the electron-donor parameter (*γ^–^*) **(b)**. Data are presented by three independent biological replicates as mean ± SD.


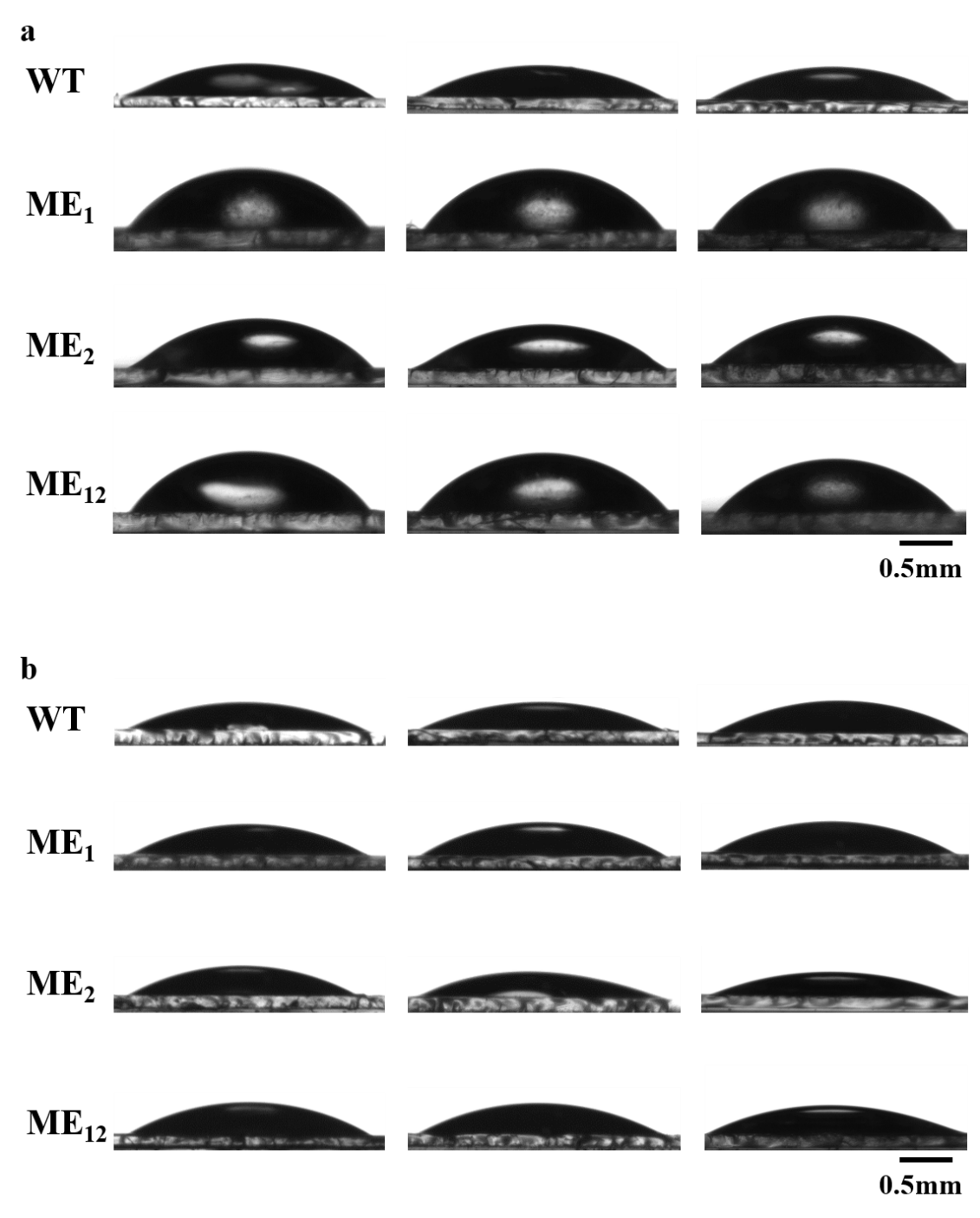


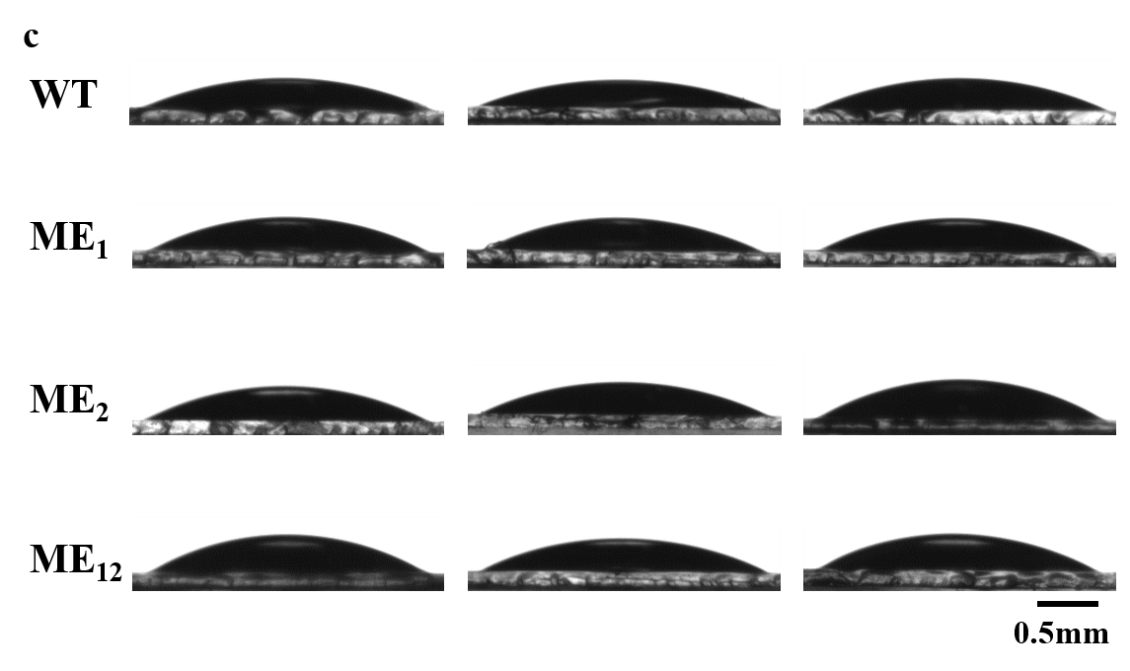


**Fig. S5 Images of contact angles.** Images of contact angles measured by dropping a drop of probe liquids, including water **(a)**, formamide **(b)**, and diiodomethane **(c)**, onto the surface of bacterial cells of different *S. oneidensis* recombinant strains with disruption of *exeS* and *exeM* genes encoding cell surface polysaccharide-biosynthesis enzymes individually and in combination, respectively.

**
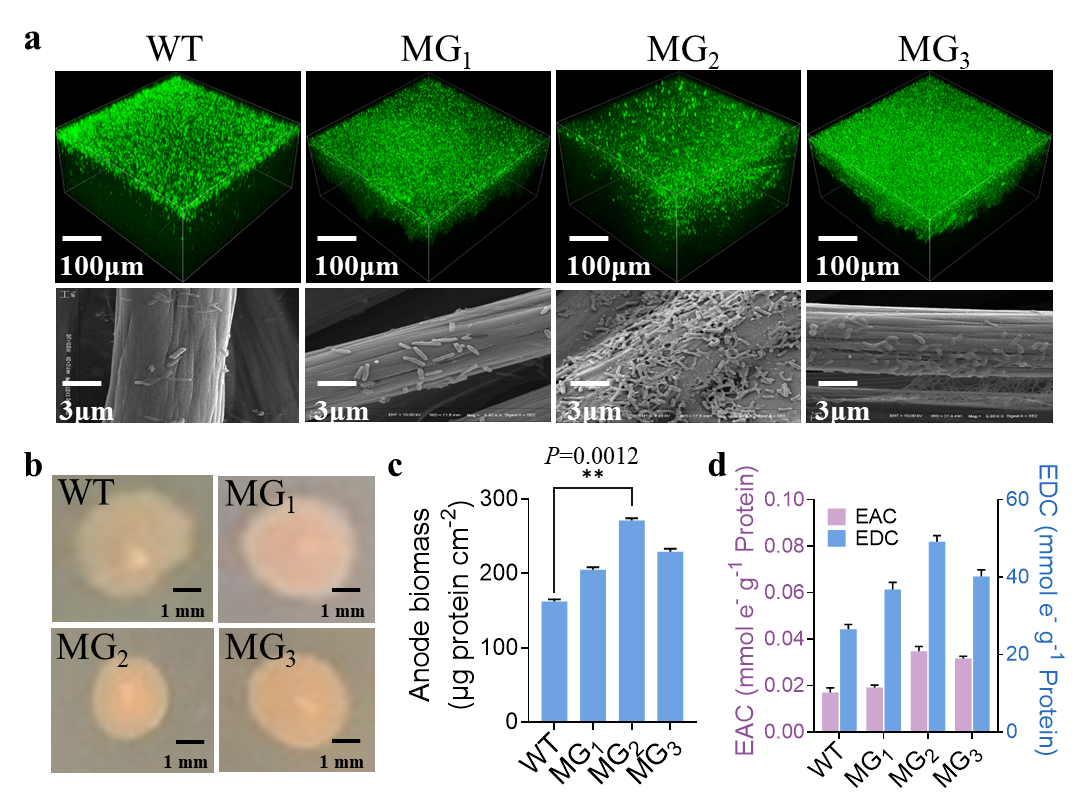
**

**Fig. S6 Increasing vertical extension of biofilm. a)** CLSM (top) and SEM (bottom) images of anode biofilms equipped with strains MG_1_, MG_2_, and MG_3_. **b)** Colony morphologies of the strains. **c)** Anode biomass of biofilm formed by the strains in MFCs. **d)** EAC and EDC of the strains in MFCs. Data are presented by three independent biological replicates as mean ± SD.


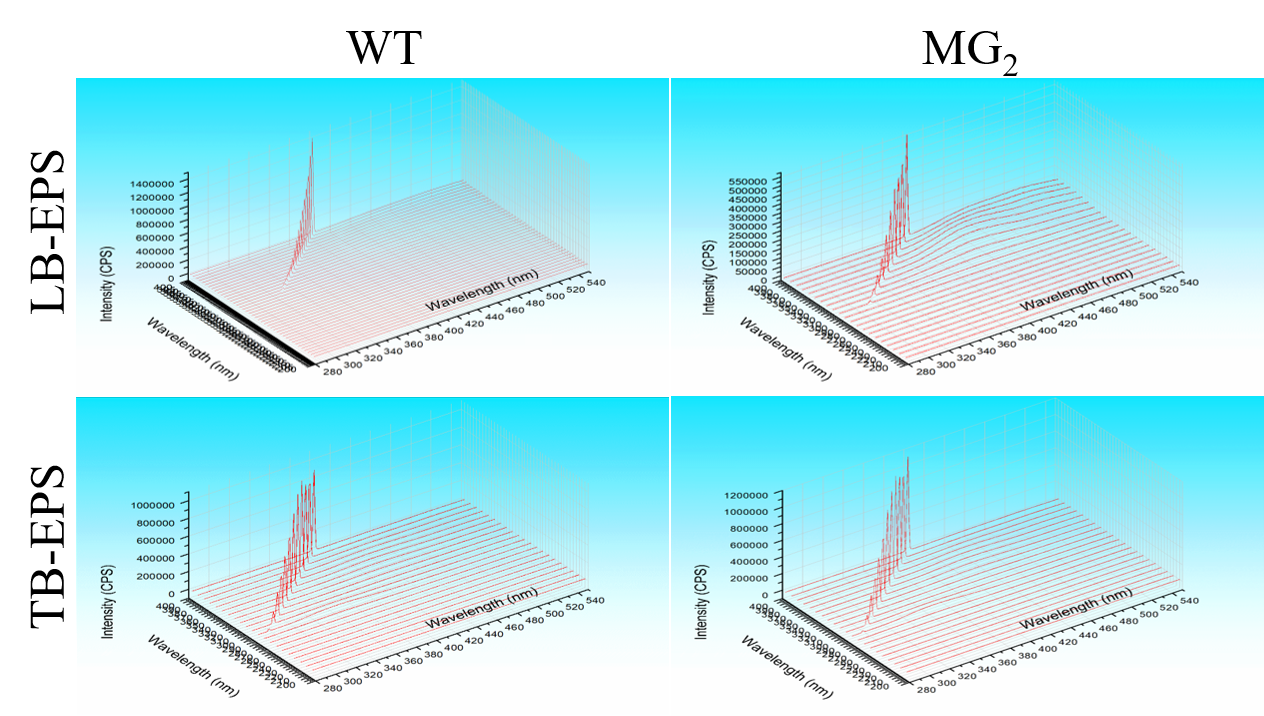


**Fig. S7 Three-dimensional fluorescence spectroscopy analyses.** Three-dimensional excitation–emission matrix fluorescence spectroscopy analyses of tight-bound EPS (TB-EPS) and loose-bound EPS (LB-EPS) of anode biofilms with *S. oneidensis* recombinant strains.

**Fig. S8 Genes assembly of recombinant strains.** A depiction of *S. oneidensis* recombinant strains that assembled genes from initial contact stage (*so317*1), adhesion stage (*exeS*), and growth stage (so*1942* and *so3491*), resulting in two *S. oneidensis* recombinant strains SG (*S. oneidensis so317*1^-^*so1942*^-^*so3491*^-^) and SEG (*S. oneidensis* *so3171^-^**exeS^-^so1942^-^so3491^-^*), respectively. And an antisense RNAs (asRNAs) was expressed in SG for down-regulating gene *exeS*, resulting in recombinant strain SE^r^G.


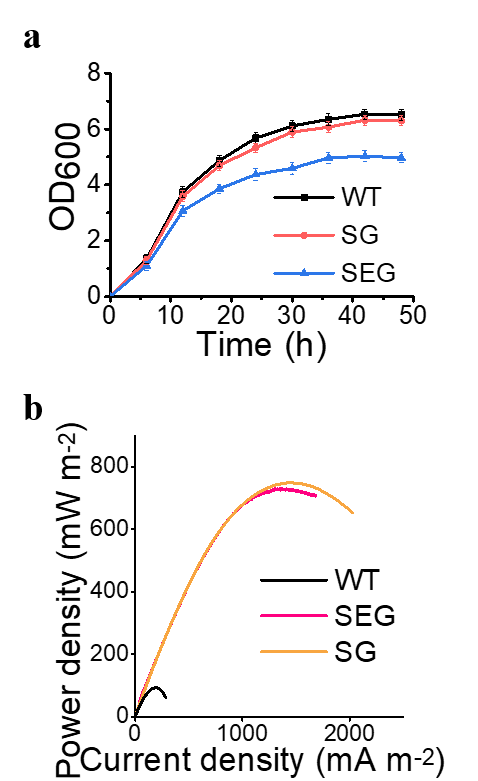


**Fig. S9 Growth and electricity production of recombinant strains. a)** Aerobic growth curve inoculated with WT and *S. oneidensis* recombinant strains SG and SEG, respectively. **b)** Output power density curves in MFCs of WT and *S. oneidensis* recombinant strains SG and SEG, respectively. Data are presented by three independent biological replicates as mean ± SD.


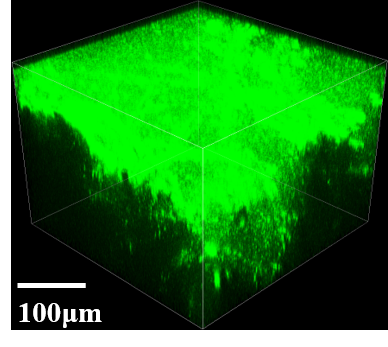


**Fig. S10 CLSM images.** CLSM images of aggregations appeared in the rG/SE^r^GC3F artificial biofilm.


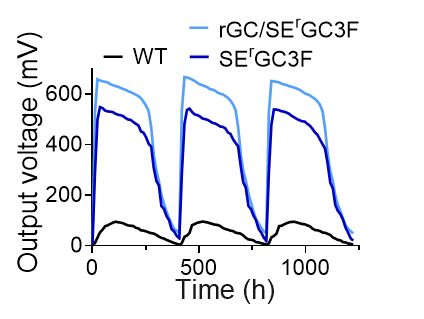


**Fig. S11 Multi-cycle voltage output of the strains.** Multi-cycle voltage output of SE^r^GC3F (dark blue line), artificial biofilm rGC/SE^r^GC3F (sky blue line), and the WT (black line) in MFCs. Data are presented by three independent biological replicates as mean ± SD.

**Table S1. Summary of the reported performances of MFCs with engineered *S. oneidensis*.**

| **Anode** | **Carbon source** | **Modification** | **Power Density**  **(W m^-2^)** | **Ref.** |
| --- | --- | --- | --- | --- |
| Graphite felt | glucose | Introducing the glucokinase (*glk*) and glucose facilitator (*glf*) genes from *Z. mobiliz* to *S. oneidensis.* | * | *(80)* |
| Graphite carbon | lactate | Characterize a native pathway for inducing gene expression with trimethylamine *N*-oxide in *S. oneidensis*. | * | *(81)* |
| Carbon cloth | xylose | An engineered *S. oneidensis* with xylose as sole carbon source directly used for bioelectricity production by assembling intracellular xylose metabolic pathways with xylose transporters. | 0.002 | *(22)* |
| Carbon felt | lactate | Bioelectricity production was improved by enhancing mediator synthesis and strengthening metal-reducing conduits. | 0.037 | *(21)* |
| Stainless steel | lactate | To bind cell with the SS electrode, the curli nanofibers on cell surface was designed with a metal-binding domain. | 0.055 | *(82)* |
| Graphite felt | lactate | Bioelectricity production was improved by disrupting the synthesis of cell surface polysaccharide*.* | 0.065 | *(57)* |
| Graphite felt | lactate | Screening mutants *so1860* from a random transposon insertion library for altered colony morphology. | 0.087 | *(83)* |
| Carbon felt | lactate | The EET of *S. oneidensis* MR‐1 was promoted by elevating intracellular cAMP level. | 0.096 | *(86)* |
| Carbon cloth | lactate | The regeneration of intracellular NADH was enhanced by driving the metabolic flux with modular metabolic engineering strategy. | 0.106 | *(85)* |
| Graphite felt | lactate | To adapt to electrode-respiring, a mutant was constructed by knocking out gene *so3350*. | ~0.11 | *(86)* |
| Carbon cloth | lactate | Enhancing biofilm formation and bioelectricity generation by overexpressing of gene *yedH* including in c-di-GMP biosynthesis pathway*.* | 0.16 | *(87)* |
| Carbon cloth | lactate | The metabolic fluxes of de novo, salvage, universal biosynthesis were redirected towards NAD^+^ bio-synthesis using a modular synthetic biology strategy. | 0.162 | *(7)* |
| Carbon cloth | lactate | Enhancing bioelectricity production by constructing an artificial conductive anode biofilm of *Shewanella*-inoculated MFCs. | 0.21 | *(88)* |
| Carbon cloth | lactate | Enhancing electron transfer by a synthetic flavin pathway. | 0.22 | *(69)* |
| Carbon cloth | lactate | Overexpressing the NDH II enzyme ndh II (Gene ID: 4921489) from *S. loihica* PV-4 for enhancing electron trans-IM movement in *S. oneidensis* MR-1 strain. | 0.37 | *(89)* |
| Carbon cloth | lactate | Enhancing electron transfer by increasing flavins synthesized by a synthetic riboflavin pathway and OprF pore protein in the mutant *S. oneidensis* CP2-1-S1. | 1.12 | *(13)* |
| **Carbon cotton** | **lactate** | **Enhancing biofilm formation and bioelectricity generation by full-cycle physiological regulation of electroactive biofilm.** | **3.62** | **This study** |

* The current density and power density were not described in the published articles. However, as characterized in these articles, the performance of these engineered *S. oneidensis* did not improve significantly compared with the wild-type *S. oneidensis*.

**Table S2. Contact angles (*θ*, °) of *S. oneidensis* strains. W, water; FO, formamide; DIM, diiodomethane. Data are presented by three independent biological replicates as mean ± SD.**

| **Strains** | ***θ*_W_** | ***θ*_FO_** | ***θ*_DIM_** |
| --- | --- | --- | --- |
| MS_1_ | 45.2 ± 0.9 | - | - |
| MS_2_ | 62.5 ± 0.6 | - | - |
| MS_12_ | 48.5 ± 0.8 | - | - |
| ME_1_ | 51.5 ± 0.7 | 30.9 ± 0.6 | 22.7 ± 0.8 |
| ME_2_ | 39.5 ± 0.6 | 25.2 ± 0.8 | 26.6 ± 0.5 |
| ME_12_ | 44.3 ± 0.8 | 28.5 ± 0.6 | 27.4 ± 0.7 |
| WT | 30.1 ± 0.5 | 26.1 ± 0.9 | 23.3 ± 0.9 |

**Table S3. Surface tension of *S. oneidensis* strains and the surface free energy (ΔG_bwb_) between cells (b) in water (w). Data are presented by three independent biological replicates as mean ± SD.**

| **Strains** | **Surface-tension component (mJ m^-2^)** | | | | | **ΔG_bwb_**  **(mJ m^-2^)** |
| --- | --- | --- | --- | --- | --- | --- |
|  | ***γ^LW^*** | ***γ^+^*** | ***γ^–^*** | ***γ^AB^*** | ***γ^Total^*** |  |
| WT | 46.8 ± 0.4 | 0.1 ± 0.8 | 46.3 ± 0.9 | 4.5 ± 0.6 | 51.3 ± 0.8 | 23.5 ± 0.9 |
| ME_1_ | 47.2 ± 0.6 | 0.4 ± 0.5 | 22.5 ± 0.9 | 5.7 ± 0.9 | 52.9 ± 0.7 | -15.2 ± 0.9 |
| ME_2_ | 45.8 ± 0.9 | 0.3 ± 0.8 | 35.9 ± 0.7 | 6.3 ± 0.8 | 52.1 ± 0.6 | 8.2 ± 0.6 |
| ME_12_ | 45.4 ± 0.8 | 0.6 ± 0.7 | 29.1 ± 0.7 | 8.3 ± 0.9 | 53.7 ± 0.9 | -2.7 ± 0.8 |

**Table S4. Surface tension parameters of the probe liquids *(58)*.**

| **Liquid** | ***γ^LW^*** | ***γ^+^*** | ***γ^–^*** | ***γ^AB^*** | ***γ^Total^*** |
| --- | --- | --- | --- | --- | --- |
| Water | 21.8 | 25.5 | 25.5 | 51.0 | 72.8 |
| Formamide | 39.0 | 2.28 | 39.6 | 19.0 | 58.0 |
| Diiodomethane | 50.8 | 0 | 0 | 0 | 50.8 |

**Table S5. Primers used in RT-qPCR.**

| **qPCR primer name** | **Sequences (5’ to 3’)** |
| --- | --- |
| *gyrB*-F | GGAACGACGGCTACCAAGA |
| *gyrB*-R | GTCAACGCACTACGGAAACC |
| *motX*-F | CCGTTTGATAATACTCGGCAAG |
| *motX*-R | GGAAATGCTCAATCACGGAAC |
| *motA*-F | TATCATAAATGCAGGTAGGTCT |
| *motA*-R | TTTTATAGGTGTAATTGTTGCG |
| *flrA*-F | ATTGTGGGTAGTTTAATGGCTCT |
| *flrA*-R | CAGTATCCTTGGCAACCGATT |
| *flaA*-F | TGATATTATGTGCTAAGCGGTTT |
| *flaA*-R | CTTTATCAGTAGGTGCGTTG |
| *pilT*-F | TTTCTTAATCAGAGTTTGCGAGA |
| *pilT*-R | GGCAGCTAAGACTATCGACC |
| *pilY*-F | ATTACCGCTTTTACTTGCC |
| *pilY*-R | CCTATGTGATTTATGTTACCGAT |
| *fimV*-F | CTTCTGCAGTATCTTGGGCAT |
| *fimV*-R | CCATTGCTGCCGAATTGGAA |

**Table S6. Summary of the genes used in this study.**

| **Function** | **Gene name** | **Genbank** | **Source** |
| --- | --- | --- | --- |
| Extracellular protein-biosynthesis enzymes genes | *aggA* | AAN57289.1 | *S. oneidensis* |
|  | *rbmA* | AWK28776.1 | *Vibrio cholerae* |
|  | *saeP* | CAC8329192.1 | *Staphylococcus aureus* |
| Cell surface polysaccharide-biosynthesis enzymes genes | *so_1860* | AAN54912.1 | *S. oneidensis* |
|  | *so_3171* | AAN56171.1 | *S. oneidensis* |
| Extracellular endonucleases genes | *exeS* | CP053946.1 | *S. oneidensis* |
|  | *exeM* | AE014299.2 | *S. oneidensis* |
| c-di-GMP hydrolase genes | *so_1942* | AAN54993.1 | *S. oneidensis* |
|  | *so_3491* | AAN56484.1 | *S. oneidensis* |
|  | *so_4711* | AAN57670.1 | *S. oneidensis* |
| OM *c*-Cyts | *omcC* | UAC03421.1 | *Geobacter sulfurreducens* |
|  | *omcE* | AAR33949.1 | *Geobacter sulfurreducens* |
|  | *mtoA* | ADE12722.1 | *Sideroxydans lithotrophicus* |
| Flavin biosynthesis genes | *ribA* | CAB14258.1 | *B. subtilis* |
|  | *ribC* | CAB13540.1 | *B. subtilis* |
|  | *ribD* | CAB14260.1 | *B. subtilis* |
|  | *ribE* | CAB14259.1 | *B. subtilis* |
|  | *ribH* | CAB14257.1 | *B. subtilis* |

**Table S7. The synthesized gene sequences in this study.**

| **Genes** | **Sequences** |
| --- | --- |
| *rbmA* | ATGAAAAAATTAATCATCGCCTTATTAAGCGGTAGCATCGCCTTAAGCACTAGCTTAAGCGTGTTTGCCGTGGAAAACCAAATGGCCAACGAAGAATTTTTATTAGATGCCCAAATCCAATTAAGCAAAAACCAAGTGCCAACCGAGGGTGGCTACTTAAGCGCGAGCATCGATTTAACCAACATCGGTGATGGTGATGCGAGCTTAAAATACTGGGTGAGCGTGAAAGGTCCAAAAGGTATCGTGTTTCCTGCCAAAAGCGTGGTGGGTGTGAACAGCAGCGAATTTGATAGCGAAAACATCGAAGAAGGTAGCGCCTTACACATCGAACGTGGTATCTGGGTGCGTGAATACATGGATGATGGTTTATACCAAGTGGCGGTGGAAGGTGTGAACGTGGAAACCGGTAAAACCTTTAGCAAAAGCGAAACCTTTGCCAAAGGTGTGAGCATCGAACAACCTGCCGCCATCGATGGTTTAATCTTAGAAGCCTTTGCCGTGAACGATACCATCTTTCCAAGCGAAGGTGGTTACTTAATCCTGGATTTACAAGCCCACAACACCCGTGATCAAAGCGCCAACATCGAATTTTGGGTGACCGCCGTGGGTCCTGATGGTTTAGCCATCCCTGTGCACGCCCGTGTGTTAAAAAGCGTGCCTGCCTTAGAAGAATTAAGCATCATCCGTGGTTTTACCTTAGATGCGAGCTACCCTGATGGTGAATACACCATCGTGCCACAATTATACGATGTGGATAGCGGTAAACGTGTGGAGGGTGCGATCAAAGTGTACAAAGGTGAACGTAGCGTGACCGATTAA |
| *saeP* | ATGAAAATCAAAACCTTACTGTTAAGCGGTACCATCGCCACTAGCTTACTGTTAGGTGCCTGTAACAACATGGATGATAAAAAAGATAGCATGAAAGAAGAAACCAAAAGCGAAAGCAAAATGGAAAACAAAGATATGGATAACGCCAAAGTGATGAAAGAAGGTATGTTTAAAGGTGAAAACAAAGAAAAAGTGGAAGGTAAAGCCATGATCAAAGATGGTAAATTAATGTTAAAAGATTTTAGCTCGAGCAAAGGTCCTGATTTACACGTGTACTTAACCAAAGATGGTGATATCAAAAAAGGTAAAAAAATCGATATGGTGGATTACGCCAAAAGCGAACAAAGCTTTGATTTAAAAGGTGTGAACGTGAAAGATTACAACACCGTGACCATCTACTGTGATAAAGCCCACGTGACCTTTGGTAGCGCCATGTTAAAATAA |
| *omcC* | ATGTCTCGTAAAGTTACTAAATACTCTGCTGTTCTCGCTGTGAGCTTATTCGCTGCTGCTTTAGCTGGTTGTGGTTCTGAAAACAAAGAAGGTACTGTTGGTACTGGTCCAGGTGGTGTTGCTACTGTTGGTGATACTGCTTGTGTTCAATGTCACTCTGCTGTTGTTGATCCATTAACTGGTGAATCTATCATCACTCAATACACTCGTTCTTTCCACTACTCTAAAGGTGTTGGTTGTGAAGGTTGTCACGGTGGTGGTGCTCAACACAACGGTGTTGGTCCATTACCATTCCCATTAGCTGGTCAATCTGAAGCTCAAATCGCTGCTCGTTGTGCTTCTTGTCACAACGGTGTTATCGCTCCATTATCTTCTTCTCCAAACTTCGTTAACGGTAACCACGCTAACCCATTCGGTGGTGAAGAAGCTAAAGAAAACTTATGTTCTCGTTGTCACTCTCACGAAGGTGCTATCTTCGGTGCTCAAGCTGGTTTCACTGGTGATGGTAACATCTTACGTAACGCTGCTTACCAGCCAGTATACCCACAAGACCCAGAAACTTTCAACGTTATGACTTGTGCTACTTGTCACCAACACGGTGGTGCTCAACGTCAAGTTTTCACTCAAATCTCTACTGCTGGTGTTCCAAACTCTCGTCGTACTGTTGCTTGGGACCCAAACCGTAACTCTATCAACGATCAATACGATTTATGTACTTCTTGTCACACTGTTAACACTATGACTGGTACTTTAATCGGTTCTGGTAACGTTTTACAAATCTTCACTTCTAACGCTGTTGGTTCTGGTACTAAATCTGTTACTACTGCTCCATTCTACCACAACACTCGTTGGTTCCGTACTTTACCATCTACTCACTACGATTTCCCAGAATCTAAAACTACTGCTTCTGGTACTACTATCGAAGGTTACGTTATCCGTCGTAACACTGCTAACCCATGTTTCGATTGTCACGGTCACGAATTTCAAACTAACACTCGTCGTTTAGCTGGTGCTGATCGTCCAAACACTATCTTCTTAGATTGGGGTCAATCTGCTCACGGTGGTAAATTATTACAAGCTAAAGTTGCTGCTGCTGCTCTCGCTTCTTCTGGCGCTGCTGAAGTTGATGATGTTATGAAAGCTGGTGCTACTGATGCTACTGCTCCAGGTTGGACTCACTACAACTGGGATGATACTGCTTCTCGTGGTGCTTGTCAACGTTGTCACACTTCTACTGGTGCTTCTAACTTCTTAAACAACCCAGCTGGTTACGATCGTACTGGTGCTGGTAACTCTTTCACTCACTTAGCTGGTTGGACTTCTTCTAACAAACGTTCTGATCAAAACGAATTATTATACTGTTGGGGTTGTCACACTAAAGCTGGTACTGGTGAATTACGTAACCCAGGTGCTATCACTGAAGTTTACCCAGGTATCAACTCTACTTCTACTGGTACTACTGGTTTAGATGTTACTGTTTCTTACCCAGATATCAAAGGTTCTAACGTTTGTATGGGTTGTCACTTAGGTCGTGAAGTTGGTGATAACATCAAAGCTATCACTGATGCTGATGGTATCTTAGGTTTCGTTAACTCTCACTACTTAACTGCTGGTGGTCAATTATTCGGTACTACTGGTTACGAATACGCTACTCGTTCTTACGCTAACCCAGCTTTCTTCCAACACGATAAAATCGGTACTGCTGCTGCTCCAGGTACTGGTACTAACGGTCCATGTGCTGGTTGTCACATGACTACTCCAACTTCTCACTTATTCTTACCAGTTACTAAAGATGGTACTGGTGCTATCACTGCTATCACTTCTACTGCTTGTGTTACTTGTCACGCTGGTACTTTCGCTTTAACTCCAGAAGGTTTAACTGCTGAAGAAGAAGAATACGTTGCTAGTCTCGAAGCTCTCAAAGCTGCTCTCGCTGGTAAAGGTATCTTATTCTTCAACGCTCACCCATACTTCTACCGTGATACTAACGCTAACGGTATCGCTGATCCAGGTGAAACTGTTTCTTCTAACGCTTTCACTAACTGGGCGGGTGTCTACGGATTAGCTTTATGGCAAGATGTTATGGGTGCTGCTTTCAACGCTAACTTATTAATCCACGATCCAGGTGGTTACGCTCACAACCGTTTCTACTCGAAGCGTCTCATCTGGGATTCGATCGATTTCATCTTTGACGGTGTGCTAAACAACGATGTTACTGCTGCTATCGATGCTCAAGTTACTGCTGCTCGTTTAGATTCTGCTACTGCTACTGCTGCTAAAGCTTACTTAGGTGCTACTCGTCCATAA |
| *omcE* | ATGCGTTCTGAAGTTAAAATCGGTTTAGCTTTAACTGCTTTATTAGTTGCTGTTACTGCTGCTGGTGCTGCTTCTATCAAAAACACTAAACACGATTTATCTTCTGGTTCTACTGGTGCTACTTTCAAAGCTACTAACACTGATCAGATATGTGTGTTCTGCCACACTCCACACAACGCTCAACAAGATATCCCATTATGGAACCGTGGTAACCCAACTGCTTCTACTTTCACTTTATACTCTTCTTCTTCTATGAACAACGTTCCAGTTAAACAAGGTTTCACTGCTGATTCTATCTCTTTATTCTGTATGTCTTGTCACGATGGTGCTACTGGTTTAGGTGGTGCTGTTCACAACGATCCAAACGGTGCTGCTATCGCTATGGTTGGTGGTAACGATTTAATCACTGGTGAAGCTAACTTAGGTACTGATTTATCTAACGATCACCCAGTTAACTTCGAAGTTACTCCAGCTGGTATCGCTGCTGATGGTAACTTAGGTGCTTTAGATACTGGTACTAACCCACCAACTATGAAAACTGGTGATGTTACTAACGGTTTACCATTATTCAAATCTGCTCGTGGTGCGACAACTCTCGAATGTGGTTCGTGTCACAAAGTTCACGATAACACTGATGCTCCATTCTTACGTACTACTATGGCTGGTTCTAAATTATGTTTAGGTTGTCACAAAAAATAA |
| *mtoA* | ATGAAACCATTACGTAAAATCATGGTTTTATCTGCTTTCATCACTGGGATGGCTGCGATGCCATGCCTAATGGCTGCTGATGAACAACCAGCTGCTCAATCTGCTCCAGCTGCTGAAGCTCAACCAGCTTCTCAATCTCAATTACCAGATTTATCTATGGAAGCTAAAGCTCCACAAACTGCTCAAGAATCTTTAAAACGTGATGCTGTTTGTACTCGTTGTCACGATGAATCTGAAACTACTCCAATCTTAGCTATCTACCAAACTAAACACGGTTTCCGTGGTGATATGCGTACTCCAAACTGTCAAACTTGTCACGGTGAATCTGCTAACCACTTAAAAGGTAACGTTGATGGTAAAGGTCGTCCAGCTCCAGATGTTGTTTTCAAGAAGCACACATTCCCAGCTTCTGACGATAAAGTTCGTTCTGCTCAATGTTTAACTTGTCACAAAGGTACTAACCGTACTAACTGGGCTGGTTCTGCTCACCAATCTAACCAAATGGCTTGTAACGATTGTCACAAAATCCACGCTAAAGCTGATACTGTTCGTGAACGTGCTACTCAAACTGAAGTTTGTTACACTTGTCACAAAGAACGTCGTGCTGATGCTCACAAAATCTCTACTCACCCAATCGAAGCTGGTAAAGTTGTTTGTTCTGATTGTCACAACCCACACGGTTCTGCTGGTCCAAAATTATTAAAAAAAAACACTGTTACTGAAACTTGTTTCACTTGTCACGCTGATAAACGTGGTCCATTCTTATTCGCTCACCAACCAGTTACTGAAGATTGTACTAACTGTCACATGCCACACGGTTCTAACATCGCTCCATTATTAAAAACTCGTCCACCATTCATGTGTCAAGAATGTCACGATGGTTCTCACGCTTCTGGTACTGCTGTTGGTCCAAACGCTGCTGGTTACCAGGCGGGCTTATCTACTATCAATGCTGCTGGTACTGGTGCTTTATACCCATCTGCTAACAACGTTGGTAACGCTTGTATGAACTGTCACCGTCAAATCCACGGTTCTAACTCTCCAGCTGGTGGTTACTTACAACGTTAA |
| *ribA* | ATGTTCCACCCAATCGAAGAAGCTTTAGATGCTTTAAAAAAAGGTGAAGTTATCATCGTTGTTGATGATGAAGATCGTGAAAACGAAGGTGATTTCGTTGCTTTAGCTGAACACGCTACTCCAGAAGTTATCAACTTCATGGCTACTCACGGTCGTGGTTTAATCTGTACTCCATTATCTGAAGAAATCGCTGATCGTTTAGATTTACACCCAATGGTTGAACACAACACTGATTCTCACCACACTGCTTTCACTGTTTCTATCGATCACCGTGAAACTAAAACTGGTATCTCTGCTCAAGAACGTTCTTTCACTGTTCAAGCTTTATTAGATTCTAAATCTGTTCCATCTGATTTCCAACGTCCAGGTCACATCTTCCCATTAATCGCTAAAAAAGGTGGTGTTTTAAAACGTGCTGGTCACACTGAAGCTGCTGTTGATTTAGCTGAAGCTTGTGGTTCTCCAGGTGCTGGTGTTATCTGTGAAATCATGAACGAAGATGGTACTATGGCTCGTGTTCCAGAATTAATCGAAATCGCTAAAAAACACCAATTAAAAATGATCACTATCAAAGATTTAATCCAATACCGTTACAACTTAACTACTTTAGTTGAACGTGAAGTTGATATCACTTTACCAACTGATTTCGGTACTTTCAAAGTTTACGGTTACACTAACGAAGTTGATGGTAAAGAACACGTTGCTTTCGTTATGGGTGATGTTCCATTCGGTGAAGAACCAGTTTTAGTTCGTGTTCACTCTGAATGTTTAACTGGTGATGTTTTCGGTTCTCACCGTTGTGATTGTGGTCCACAATTACACGCTGCTTTAAACCAAATCGCTGCTGAAGGTCGTGGTGTTTTATTATACTTACGTCAAGAAGGTCGTGGTATCGGTTTAATCAACAAATTAAAAGCTTACAAATTACAAGAACAAGGTTACGATACTGTTGAAGCTAACGAAGCTTTAGGTTTCTTACCAGATTTACGTAACTACGGTATCGGTGCTCAAATCTTACGTGATTTAGGTGTTCGTAACATGAAATTATTAACTAACAACCCACGTAAAATCGCTGGTTTAGAAGGTTACGGTTTATCTATCTCTGAACGTGTTCCATTACAAATGGAAGCTAAAGAACACAACAAAAAATACTTACAAACTAAAATGAACAAATTAGGTCACTTATTACACTTCTAA |
| *ribC* | GTGAAAACTATCCACATCACTCACCCACACCACTTAATCAAAGAAGAACAAGCTAAATCTGTTATGGCTTTAGGTTACTTCGATGGTGTTCACTTAGGTCACCAAAAAGTTATCGGTACTGCTAAACAAATCGCTGAAGAAAAAGGTTTAACTTTAGCTGTTATGACTTTCCACCCACACCCATCTCACGTTTTAGGTCGTGATAAAGAACCAAAAGATTTAATCACTCCATTAGAAGATAAAATCAACCAAATCGAACAATTAGGTACTGAAGTTTTATACGTTGTTAAATTCAACGAAGTTTTCGCTTCTTTATCTCCAAAACAATTCATCGATCAATACATCATCGGTTTAAACGTTCAACACGCTGTTGCTGGTTTCGATTTCACTTACGGTAAATACGGTAAAGGTACTATGAAAACTATGCCAGATGATTTAGATGGTAAAGCTGGTTGTACTATGGTTGAAAAATTAACTGAACAAGATAAAAAAATCTCTTCTTCTTACATCCGTACTGCTTTACAAAACGGTGATGTTGAATTAGCTAACGTTTTATTAGGTCAACCATACTTCATCAAAGGTATCGTTATCCACGGTGATAAACGTGGTCGTACTATCGGTTTCCCAACTGCTAACGTTGGTTTAAACAACTCTTACATCGTTCCACCAACTGGTGTTTACGCTGTTAAAGCTGAAGTTAACGGTGAAGTTTACAACGGTGTTTGTAACATCGGTTACAAACCAACTTTCTACGAAAAACGTCCAGAACAACCATCTATCGAAGTTAACTTATTCGATTTCAACCAAGAAGTTTACGGTGCTGCTATCAAAATCGAATGGTACAAACGTATCCGTTCTGAACGTAAATTCAACGGTATCAAAGAATTAACTGAACAAATCGAAAAAGATAAACAAGAAGCTATCCGTTACTTCTCTAACTTACGTAAATAA |
| *ribD* | ATGGAAGAATACTACATGAAATTAGCTTTAGATTTAGCTAAACAAGGTGAAGGTCAAACTGAATCTAACCCATTAGTTGGTGCTGTTGTTGTTAAAGATGGTCAAATCGTTGGTATGGGTGCTCACTTAAAATACGGTGAAGCTCACGCTGAAGTTCACGCTATCCACATGGCTGGTGCTCACGCTGAAGGTGCTGATATCTACGTTACTTTAGAACCATGTTCTCACTACGGTAAAACTCCACCATGTGCTGAATTAATCATCAACTCTGGTATCAAACGTGTTTTCGTTGCTATGCGTGATCCAAACCCATTAGTTGCTGGTCGTGGTATCTCTATGATGAAAGAAGCTGGTATCGAAGTTCGTGAAGGTATCTTAGCTGATCAAGCTGAACGTTTAAACGAAAAATTCTTACACTTCATGCGTACTGGTTTACCATACGTTACTTTAAAAGCTGCTGCTTCTTTAGATGGTAAAATCGCTACTTCTACTGGTGATTCTAAATGGATCACTTCTGAAGCTGCTCGTCAAGATGCTCAACAATACCGTAAAACTCACCAATCTATCTTAGTTGGTGTTGGTACTGTTAAAGCTGATAACCCATCTTTAACTTGTCGTTTACCAAACGTTACTAAACAACCAGTTCGTGTTATCTTAGATACTGTTTTATCTATCCCAGAAGATGCTAAAGTTATCTGTGATCAAATCGCTCCAACTTGGATCTTCACTACTGCTCGTGCTGATGAAGAAAAAAAAAAACGTTTATCTGCTTTCGGTGTTAACATCTTCACTTTAGAAACTGAACGTATCCAAATCCCAGATGTTTTAAAAATCTTAGCTGAAGAAGGTATCATGTCTGTTTACGTTGAAGGTGGTTCTGCTGTTCACGGTTCTTTCGTTAAAGAAGGTTGTTTCCAAGAAATCATCTTCTACTTCGCTCCAAAATTAATCGGTGGTACTCACGCTCCATCTTTAATCTCTGGTGAAGGTTTCCAATCTATGAAAGATGTTCCATTATTACAATTCACTGATATCACTCAAATCGGTCGTGATATCAAATTAACTGCTAAACCAACTAAAGAATAA |
| *ribE* | ATGTTCACTGGTATCATCGAAGAAACTGGTACTATCGAATCTATGAAAAAAGCTGGTCACGCTATGGCTTTAACTATCAAATGTTCTAAAATCTTAGAAGATGTTCACTTAGGTGATTCTATCGCTGTTAACGGTATCTGTTTAACTGTTACTGATTTCACTAAAAACCAATTCACTGTTGATGTTATGCCAGAAACTGTTAAAGCTACTTCTTTAAACGATTTAACTAAAGGTTCTAAAGTTAACTTAGAACGTGCTATGGCTGCTAACGGTCGTTTCGGTGGTCACTTCGTTTCTGGTCACGTTGATGGTACTGCTGAAATCACTCGTATCGAAGAAAAATCTAACGCTGTTTACTACGATTTAAAAATGGATCCATCTTTAACTAAAACTTTAGTTTTAAAAGGTTCTATCACTGTTGATGGTGTTTCTTTAACTATCTTCGGTTTAACTGAAGATACTGTTACTATCTCTTTAATCCCACACACTATCTCTGAAACTATCTTCTCTGAAAAAACTATCGGTTCTAAAGTTAACATCGAATGTGATATGATCGGTAAATACATGTACCGTTTCTTACACAAAGCTAACGAAAACAAAACTCAACAAACTATCACTAAAGCTTTCTTATCTGAAAACGGTTTCTAA |
| *ribH* | ATGAACATCATCCAAGGTAACTTAGTTGGTACTGGTTTAAAAATCGGTATCGTTGTTGGTCGTTTCAACGATTTCATCACTTCTAAATTATTATCTGGTGCTGAAGATGCTTTATTACGTCACGGTGTTGATACTAACGATATCGATGTTGCTTGGGTTCCAGGTGCTTTCGAAATCCCATTCGCTGCTAAAAAAATGGCTGAAACTAAAAAATACGATGCTATCATCACTTTAGGTACTGTTATCCGTGGTGCTACTACTCACTACGATTACGTTTGTAACGAAGCTGCTAAAGGTATCGCTCAAGCTGCTAACACTACTGGTGTTCCAGTTATCTTCGGTATCGTTACTACTGAAAACATCGAACAAGCTATCGAACGTGCTGGTACTAAAGCTGGTAACAAAGGTGTTGATTGTGCTGTTTCTGCTATCGAAATGGCTAACTTAAACCGTTCTTTCGAATAA |
